# Asymmetric distribution of mitochondrial Ca^2+^ regulators specifies compartment-specific mitochondrial function and neuronal development

**DOI:** 10.1038/s12276-026-01803-2

**Published:** 2026-08-05

**Authors:** Dong Cheol Jang, Su Yeon Kim, Won Seok Kim, In Young Choi, Eunsu Jang, Hyunsu Jung, Changuk Chung, Seokyoung Bang, Hong Nam Kim, Hae Woong Choi, Kihoon Han, Yongcheol Cho, Seok-Kyu Kwon

**Affiliations:** 1 Brain Science Institute, Korea Institute of Science and Technology (KIST), Seoul, 02792 Republic of Korea; 2 Department of Neuroscience, Korea University College of Medicine, Seoul, 02841 Republic of Korea; 3 Division of Life Sciences, Korea University, Seoul, 02841 Republic of Korea; 4 Department of Brain Sciences, Daegu Gyeongbuk Institute of Science & Technology (DGIST), Daegu, 42988 Republic of Korea; 5 Department of Biomedical Engineering, Dongguk University, Goyang, 10326 Republic of Korea; 6 Division of Bio-Medical Science & Technology, KIST School, Korea University of Science & Technology (UST), Daejeon, 34113 Republic of Korea; 7 School of Mechanical Engineering, Yonsei University, Seoul, 03722 Republic of Korea; 8 Yonsei-KIST Convergence Research Institute, Yonsei University, Seoul, 03722 Republic of Korea

**Keywords:** Cellular neuroscience, Neuronal development, Mitochondria

## Abstract

Neuronal polarization is essential for functional compartmentalization, enabling dendritic synaptic integration and axonal action potential generation. Although structural differences in mitochondria across compartments have been identified, their functional distinctions remain unclear. Here, we uncovered compartment-specific mitochondrial Ca^2+^ dynamics and their molecular determinants. In axonal mitochondria, Ca^2+^ uptake through mitochondrial Ca^2+^ uniporter occurs independently of ER-stored Ca^2+^ release, with faster matrix Ca^2+^ clearance than dendritic mitochondria, where Ca^2+^ uptake predominantly originates from ER Ca^2+^. The ER-independent mitochondrial Ca^2+^ uptake in axonal mitochondria is associated with enriched mitochondrial Ca^2+^ uniporter-regulating proteins, MICU1 and MICU2, whereas higher NCLX expression facilitates rapid Ca^2+^ clearance. Moreover, NCLX knockdown, which functionally mimics a mental retardation-associated mutation, caused more significant developmental defects of axons than dendrites in vivo, aligning with its enrichment in axons. These findings highlight fundamental Ca^2+^-modulating features and developmental importance of neuronal mitochondria in a compartment-specific manner and reveal the key underlying molecular determinants.

## Compartmentalized Mitochondrial Ca2+ Dynamics in Neurons Revealed

Neurons, with their complex structures, rely on mitochondria for energy and calcium (Ca²⁺) regulation, crucial for neuronal functions. This study explores the differences in mitochondrial Ca²⁺ regulation between axons and dendrites, focusing on cortical and hippocampal pyramidal neurons. Using genetically encoded Ca²⁺ sensors, researchers found that axonal mitochondria exhibit faster Ca²⁺ decay and are less dependent on endoplasmic reticulum (ER) Ca²⁺ release than dendritic mitochondria. This is attributed to higher levels of the Na⁺/Ca²⁺ exchanger (NCLX) and MICU1/2 proteins in axons. The study also shows that NCLX knockdown impairs axonal development but not dendritic branching, highlighting the compartment-specific roles of mitochondrial Ca²⁺ regulation. These findings suggest that compartment-specific mitochondrial Ca^2+^ dynamics are crucial for proper neuronal development and provide important insights into the mechanisms underlying neurodevelopmental disorders.

This summary was initially drafted using artificial intelligence, then revised and fact-checked by the author.

## Introduction

Neurons are among the most highly polarized cells in our body, consisting of dendrites, a soma and a unique axon, crucial for determining the flow of information propagation in the nervous system. The extreme size and morphological polarization of neurons also apply to intracellular organelles such as mitochondria that exhibit drastic structural differences in the axon and dendrite. Axonal mitochondria typically display small (~1 µm long) and spherical morphologies, whereas dendritic mitochondria adopt elongated, tubular and highly fused shapes in cortical pyramidal neurons, for example^1,2^. Moreover, they play pivotal roles in providing energy and regulating Ca^2+^ in both regions, thereby modulating various neuronal functions, including synaptic vesicle mobilization and synaptic plasticity^3–5^. Regarding mitochondrial Ca^2+^ modulation, at presynaptic sites, neurotransmitter release is influenced by mitochondrial Ca^2+^ uptake, with impaired Ca^2+^ import leading to alteration in short-term synaptic plasticity and an increase in asynchronous release^4,6–8^. In dendrites of pyramidal neurons, mitochondrial Ca^2+^ regulation also affects multiple synaptic features including excitability and spine dynamics. Despite these compartment-specific roles, whether mitochondrial Ca^2+^ regulation exhibits distinct features in axons and dendrites remains largely unexplored.

Recent studies have elucidated multiple components involved in mitochondrial Ca^2+^ influx and efflux. The mitochondrial Ca^2+^ uniporter (MCU), a channel responsible for rapid Ca^2+^ uptake, forms a complex with other subunits such as mitochondrial Ca^2+^ uptake 1 (MICU1), MICU2 or MICU3, acting as gatekeepers and modifiers of the Ca^2+^ uptake properties of MCU^9–11^. Conversely, mitochondrial Ca^2+^ clearance from the mitochondrial matrix is regulated by the Na^+^/Ca^2+^ exchanger (NCLX) under physiological conditions^12^. NCLX activity can be modulated by PKA-dependent phosphorylation^13^, and recent studies identified TMEM65 as a NCLX regulator or mitochondrial Na^+^/Ca^2+^ exchanger^14,15^. Notably, the composition of these components can vary across tissues. For example, the MICU1:MCU expression ratio is lower in heart and skeletal muscle than in the liver, leading to lower threshold and cooperativity of MCU activation^16^. Additionally, in the brain, mitochondrial Ca^2+^ regulators show distinct expression patterns depending on subregions; for instance, there is a higher level of MCU in hippocampal CA2/3 than in CA1 (refs.^17,18^). However, whether these regulatory components are differentially distributed or tuned across axonal and dendritic compartments remains unknown.

Mitochondrial dysfunction has long been implicated in neurodegenerative diseases, where defects in energy metabolism and Ca^2+^ buffering contribute to progressive neuronal loss^11,19–21^. In addition, several studies have also shown that mitochondrial defects can interfere with key processes in neuronal development, including axon branching and dendritic arborization^22,23^. Given that mitochondria serve distinct roles in axons and dendrites, their dysregulation could differentially affect these subcellular compartments depending on local functional demands. However, whether mitochondrial dysfunction divergently impacts axonal and dendritic development remains unclear.

In this study, we investigated the functional asymmetry of mitochondria in axons and dendrites regarding Ca^2+^ regulation and identified key molecular components responsible for these differences following axon isolation. Furthermore, we explored the importance of the compartment-specific functional properties of mitochondria in neuronal development.

## Methods

### Ethical statement

All experiments were performed according to protocols approved by the Korea Institute of Science and Technology Institutional Animal Care and Use Committee (IACUC) (2023-047-2) and Institutional Biosafety Committee.

### Animals

Timed pregnant CD1 females were obtained from DBL. Littermates were randomly allocated to experimental groups without consideration of their sex at the moment of ex utero electroporation (E15.5).

### Constructs

pCAG::mito (COXVIII)-YFP, pCAG::mito (COXVIII)-mTagBFP2, pCAG::mTagBFP2, pCAG::HA-mCherry and pCAG::G-CEPIA1er were previously described^1,4,24^. pCAG::mScarlet was generated by replacing IRES-GFP from pCIG2 with mScarlet. pCAG::ER-GCaMP6-150 was generated by replacing IRES-GFP from pCIG2 with ER-GcaMP6-150 (ref.^25^) (Addgene #86918). For pCAG::mito-jGCaMP8m, we inserted jGCaMP8m from pGP-CMV-jGCaMP8m^26^ (Addgene #162372) to pCAG::mito-mTagBFP2 by replacing mTagBFP2. pCAG::cyto-jGCaMP8s was generated by replacing IRES-GFP from pCIG2 with jGCaMP8s^26^ (Addgene #162371). The shNCLX-mTagBFP2 and shNCLX-Venus lentiviral short-hairpin RNA (shRNA) constructs were created from pLKO.1-TRC cloning vector^27^ (Addgene #10878). The target sequence used for shRNA knockdown of NCLX in mice was as follows: 5’-CCAGACATCTTCAGTGCTTTA-3’. We replaced the puromycin resistance sequence of scramble shRNA^28^ (Addgene #1864) with mTagBFP2 for the control vector used in shRNA experiments. Control empty vector (pCAG) was generated by deleting IRES-GFP sequence from pCIG2. pCAG::MICU1-mTagBFP2 and pCAG::MICU1-mScarlet were created by inserting MICU1 (OriGene, MR207652). pCAG::MICU2-mTagBFP2 and pCAG::MICU2-mScarlet were created by inserting MICU2 (OriGene, MR206892) into pCAG vector.

### Ex utero electroporation

A mixture of plasmid preparation (1–2 µg/µl) and 0.1% Fast Green (Sigma) was loaded in a glass capillary World Precision Instruments (WPI) and injected into the lateral ventricles of the isolated head of E15.5 mouse embryo. Then, the plasmid mixture was electroporated using an electroporator (ECM 830, BTX) with four pulses of 20 V for 100 ms with a 500-ms interval.

For cortical neuron targeting, the electroporation was carried out using gold-coated electrodes by placing the anode (positively charged electrode) on the side of DNA injection and the cathode (negatively charged electrode) on the bottom side of the head.

For hippocampal neuron targeting, the electroporation was performed using a triple-electrode setup. Two anodes were placed laterally on each the side of the head, and a single cathode was positioned rostrally at a 0° angle to the horizontal plane to target hippocampal progenitors.

### Primary neuron culture

Either embryonic mouse cerebral cortex or hippocampus was dissected in Hank’s buffered salt solution (HBSS, Invitrogen) supplemented with HEPES (10 mM, pH 7.4, Invitrogen), depending on the experimental requirements. Tissues were incubated in HBSS containing papain (Worthington) and DNaseⅠ (Sigma) and dissociated by pipetting. Dissociated cells (8.5×10^4^) were plated either on a poly-D-lysine (Sigma)/laminin (Gibco)-coated 12-mm coverslip or glass-bottomed dish (Mattek) in neurobasal media (Invitrogen) containing 2% B27, 1% glutamax, 2.5% FBS and 0.5% penicillin/streptomycin (all supplements are from Invitrogen). After 5–7 days, the medium was changed to an FBS-free supplemented neurobasal medium. Neurons were maintained for 15–21 days in vitro (DIV) in a humidified 37°C incubator with 5% CO_2_.

For Western blot experiments, dissociated cells (5.1×10^5^) were plated either on poly-D-lysine (1 mg/ml, Sigma)-coated 24-mm Transwell® with 3.0 µm Pore Polycarbonate Membrane Insert (Corning) or PDL-coated 6-well plates in neurobasal media (Invitrogen) containing 2% B27, 1% glutamax and 1% penicillin/streptomycin. Neurons were maintained for 10–11 DIV in a humidified 37°C incubator with 5% CO_2_.

### In utero electroporation

To label layer II/III cortical pyramidal neurons, in utero electroporation was done as previously described^1^. A mixture of plasmids (1–2 mg/ml) and 0.5% fast green were injected to the lateral ventricle of E15.5 CD1 mouse embryos. Electroporations were performed with tweezer-type electrodes. To label cortical progenitor cells, a positively charged part of an electrode was placed on the top of DNA injection and a negatively charged part on the opposite side of the head. Four pulses of 40 V for 50 ms were used for electroporation. Mice were sacrificed 3 weeks after birth by transcardiac perfusion as previously described^29^. Briefly, mice were anesthetized with 2.5 vol% isoflurane with 2 ml/min oxygen and perfused transcardially with 4% PFA in PBS. Isolated brains were then post-fixed for 2 h in 4% PFA and processed for further experiments. Brains were washed with PBS and sectioned using a vibratome (Leica VT1200) at a thickness of 130 μm. Sections were then washed three times with PBS and mounted on slides using VECTASHIELD Vibrance Antifade Mounting Medium (Vector laboratories).

### HEK293FT cell transfection

Human embryonic kidney (HEK) 293FT cells (Thermo Fisher) were maintained with Dulbecco’s Modified Eagle Medium (Gibco) supplemented with 10% FBS (Gibco) and 1% penicillin/streptomycin (Gibco) in a humidified 37°C incubator with 5% CO_2_. Cells were transfected via jetPrime (Polyplus) by following the manufacturer’s instructions. Three days after transfection, cells were harvested for Western blot.

### Lentiviral production and infection

Lentiviruses were produced from HEK293FT cells by co-transfection with shuttle vectors, LP1, LP2 and VSV-G. jetPrime (Polyplus) was used as the manufacturer’s protocol. 24 h after transfection, media were changed to neurobasal media, and 48 h later, supernatants were harvested and centrifuged to remove cellular debris. 100–200 μl of viral supernatants were added to primary cortical neurons at 3–5 DIV and harvested 10 days after.

### Western blotting

Proteins from whole-cell fraction and axonal fraction were separately prepared from 3-µm porous membrane. To obtain the whole-cell fraction, cold Dulbecco's phosphate-buffered saline was applied on the upper side of the membrane and cells were harvested using cell scrapers (SPL). After a brief centrifuge, the supernatant was removed, and cells were lysed using RIPA buffer (50 mM Tris-HCl pH7.4, 150 mM NaCl, 1% NP40, 0.5% sodium deoxycholate, 0.1% SDS) supplemented with the cocktail of protease inhibitor and phosphatase inhibitor at 4 °C for 1 h. Before collecting the axonal fraction, the upper side of the membrane was wiped thoroughly using a cotton swab to prevent contamination of the whole-cell fraction. The membranes were submerged in the same lysis buffer for the axonal fraction and vortexed for 5 min. After vortexing, membranes were discarded, and cell lysate was incubated at 4 °C for 1 h.

5 µg of total protein per well was loaded onto 10–12% SDS-PAGE gels and transferred to nitrocellulose membrane (Merck). After transfer, membranes were blocked for 30 min with 5% skimmed milk (LPS Solution), followed by incubation with primary antibodies overnight at 4 °C. The membranes were washed three times with 0.1% Tween20/tris-buffered saline (TBST) and incubated with HRP-conjugated secondary antibodies (Abbkine) for 1 h at room temperature, followed by three washes with TBST. The bands were detected using ECL Western blotting substrate (Amersham) and then scanned by iBright CL750 Imaging System (Thermo Fisher). Full-length Western blots are available in Supplementary Figs. 10 and 11.

The following primary antibodies were used for Western blotting in the study: MCU (Invitrogen, MA5-24702, 1:1000), MICU1 (Atlas antibodies, HPA037480, 1:1000), MICU2 (Abcam, ab101465, 1:2000), MICU3 (Invitrogen, PA5-55177, 1:2000), VDAC1 (Abcam, ab14734, 1:1000), NCLX (Abcam, ab83551, 1:1000), TOM40 (Proteintech, 18409-1-AP, 1:2000), synaptophysin (Synaptic Systems, 101011, 1:5000), histone H3 (Cell Signaling, 96C10, 1:1000), GluR2 (Sigma, MAB397, 1:1000), beta-actin (Abbkine, 1C7, 1:10000) and TMEM65 (Proteintech, 21913-1-AP, 1:500). All secondary antibodies were HRP-conjugated (Abbkine, A21020 and A21010) and used at a dilution of 1:10,000.

### Immunocytochemistry

Cells were fixed for 12 min at room temperature in 4% paraformaldehyde (PFA, T&I) with 4% sucrose (Sigma) in PBS (Sigma) and then washed three times with PBS. Samples were permeabilized with 0.2% Triton X-100 (Sigma) in PBS and incubated for 30 min with 2.5% goat serum and 0.1% BSA (Sigma) in PBS to block non-specific signals. Primary and secondary antibodies were diluted in the blocking buffer as described above. Primary antibodies were incubated overnight at 4°C, and secondary antibodies were incubated for 1 h at room temperature. Coverslips were mounted on slides with VECTASHIELD Vibrance Antifade Mounting Medium (Vector Laboratories).

The following primary antibodies were used for immunocytochemistry in the study: MCU (Novus, NBP2-76948, 1:200), HA (Biolegend, 901513, 1:500), TOM40 (Proteintech, 18409-1-AP, 1:1000), GFP (Aves lab, GFP-1020, 1:2000) and RFP (Abcam, ab62341, 1:200). All secondary antibodies were Alexa-conjugated (Invitrogen, A11003 and A21244) and used at a dilution of 1:1000.

### Analysis of callosal axon projection and dendritic branching

For analysis of callosal axon projection, brain sections at similar rostrocaudal levels were selected, and axons on the contralateral side were evaluated. Quantification was performed by measuring relative fluorescence intensity along the radial axis of the contralateral cortex. To account for variability in transfection efficiency, the fluorescence intensity of contralateral axons was normalized to that of callosal axons within the same section^1,30^. As a complementary analysis, the same contralateral cortical region of interest (ROI) was additionally quantified using area-weighted intensity (mean fluorescence intensity × covered area) after background subtraction and radial binning from white matter to pia, followed by normalization to the total signal across the full contralateral cortical ROI.

For analysis of dendritic branching, brain sections from the same mice used for axon branching analysis were examined. Transfected neurons in the ipsilateral cortex were analysed using the Simple Neurite Tracer (SNT)^31^ plugin in ImageJ. Individual dendrites were manually traced, and the number of branching points and dendritic tips was measured. Sholl analysis was performed on the same traced neurons using concentric circles spaced at 10-μm intervals to evaluate dendritic complexity relative to the distance of the soma.

For in vitro analysis of axon branching, primary cortical neurons were transfected with either shScramble or NCLX knockdown constructs using ex utero electroporation at E15.5 and fixed at 5 DIV. Axonal morphology was analysed using Fiji (ImageJ, NIH). Individual axons were manually traced, and total axon length, number of branches and longest axon length were measured as described in a previous study^30^. Branch density was calculated as the number of branches per 250 μm of axon length.

### Imaging

Fixed samples were imaged on a Nikon A1R confocal microscope (×60 objective NA 1.25). All equipment and solid-state lasers (Coherent, 405, 488, 561 and 647 nm) were controlled via Nikon Elements software. Samples were visualized by Z-stacking, which was later processed into maximum intensity projection. The MIP 2D images were then analysed using Fiji (Image J).

For live imaging, electroporated cortical neurons were imaged at 15–21 DIV with an electron multiplying charge-coupled device camera (Andor, iXon Life 897) on Nikon microscope FN1 (×60 objective NA1.0). 395, 470 and 555 nm Spectra X LED lights (Lumencor) were used for the light source. We used modified normal Tyrode’s solution as a bath solution containing the following (in mM): 145 NaCl, 2.5 KCl, 10 HEPES, 2 CaCl_2_, 1 MgCl_2_, 10 glucose, pH 7.4 at RT. The electrical stimulation is triggered by 1-ms current injections with a glass capillary electrode placed near the transfected axon, dendrite or soma. We applied 10 pulses (10 Hz) with 30 µA using a stimulator (Model 2100, A-M systems) and imaged with a 500-ms interval (2 Hz) for fluorescence signals from transfected Ca^2+^ indicators. To avoid the spontaneous activity of cultured neurons, we treated a small amount of tetrodotoxin (TTX, less than 50 nM). For blocking a sarco/endoplasmic reticulum Ca^2+^-ATPase (SERCA) pump, we treated cyclopiazonic acid (CPA, 30 μM, Tocris) for longer than 5 min. For caffeine-induced ER Ca^2+^ release, 100 mM caffeine (Sigma, C0750) was loaded into a glass capillary connected to a pressure ejection system and perfused focally onto the ROI to locally induce ER Ca^2+^ release.

For tetramethylrhodamine (TMRM, Invitrogen) imaging, cells were incubated with 50 nM TMRM for 10 min at 37°C before imaging started. To investigate changes in mitochondrial membrane potential under excitotoxic conditions, 20 μM N-Methyl-D-aspartic acid (NMDA, Sigma) was added to the bath solution. Before NMDA treatment, cells were imaged with a 500-ms interval for 5 min to assess the fluorescence baseline, then imaged for an additional 10 min after treatment.

Cell death was assessed in cultured cortical neurons at 10–12 DIV with an electron multiplying charge-coupled device camera (Andor, iXon Life 897) on a Nikon microscope FN1 (×10 objective NA0.3). Neurons were treated with 20 μM NMDA for 30 min at 37°C to induce excitotoxic stress. Imaging was performed immediately before NMDA application and repeated after treatment following three washes with normal Tyrode’s solution. The same imaging fields were revisited to identify surviving cells. Cell death was determined by counting the number of surviving GFP-expressing neurons after NMDA treatment, as described previously^32^.

### Statistics

Ca^2+^ imaging data were analysed via Nikon NIS-Elements 5.11, Fiji Image J (version 2.16/1.54p) and in-house Python scripts written in Python 3.11.2. Analyses were performed with standard scientific libraries, including NumPy 1.24.2, pandas 1.5.3, matplotlib 3.7.1 and SciPy 1.10.1. Western blot data were analysed with iBright Analysis Software (version 5.2.1 Thermo Scientific). All statistical analysis was performed using the GraphPad Prism 10 and Microsoft Excel. One- and two-way ANOVA with post hoc tests were used for group comparisons. All graphs are shown as mean ± SEM, and asterisks *, ** and *** indicate *P* < 0.05, *P* < 0.01 and *P* < 0.001, respectively. Detailed statistical methods and *n* for each experiment are written in the figure legends. For Ca^2+^ imaging experiments, one mitochondrion was analysed per axonal or dendritic process; therefore, *n* represents the number of analysed processes. For dendritic recordings, no more than two dendrites were sampled per neuron. For axonal recordings, the soma of origin could not always be unambiguously identified within the imaging field, and axonal *n* is therefore reported at the process level. All experiments were performed with at least three independent biological replicates.

## Results

### Axonal mitochondria exhibit faster Ca^2+^ decay compared with dendritic mitochondria in cortical pyramidal neurons

**Fig. 1:**
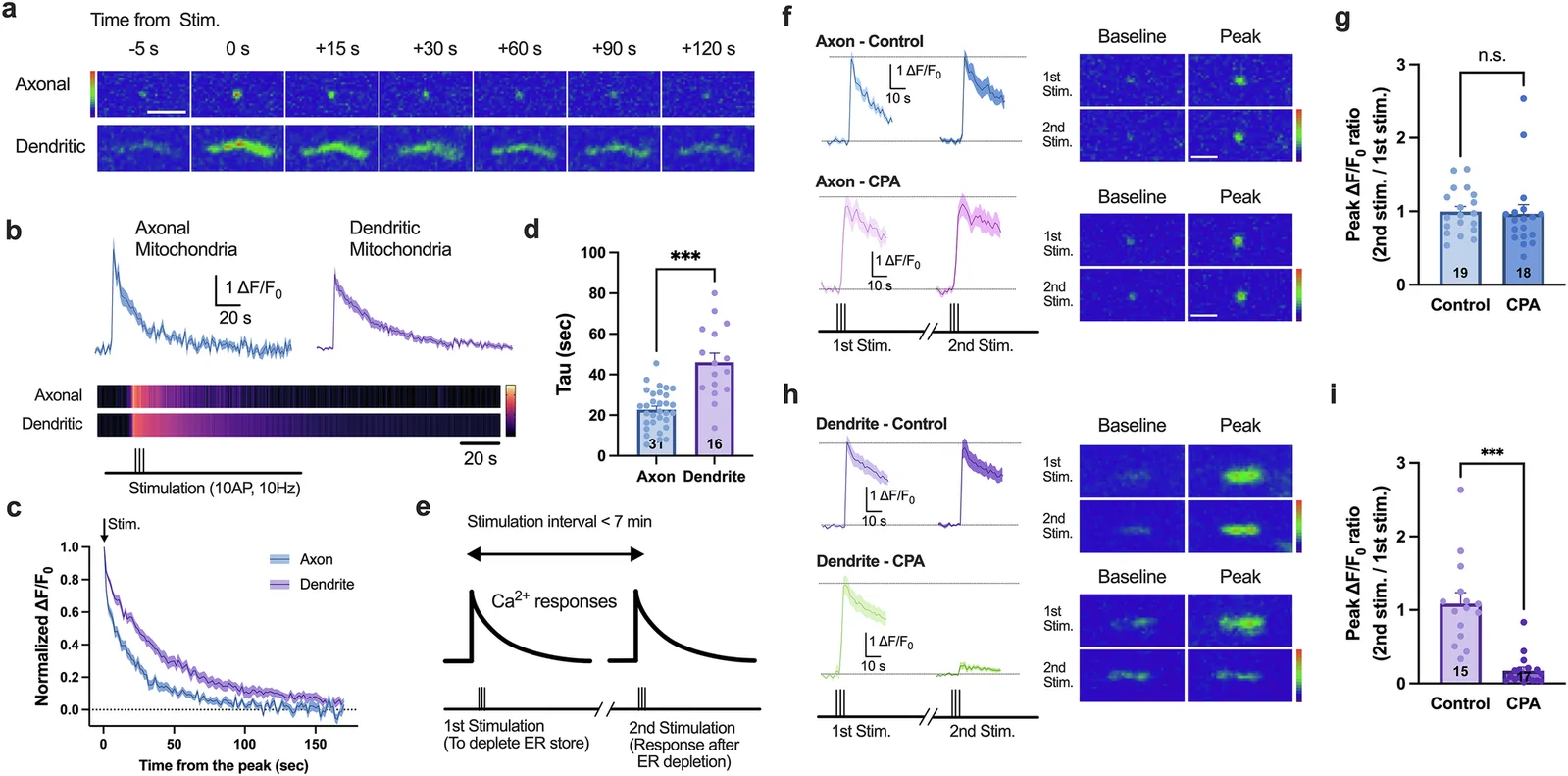
Differential Ca^2+^ decay and uptake between axonal and dendritic mitochondria in cortical pyramidal neurons. **a** Time series images from axonal and dendritic mitochondria. **b** Representative Ca^2+^ traces and line plots. The outlined regions of interest correspond to the full mitochondrial regions used for fluorescence quantification. **c** Normalized ΔF/F_0_ traces by peak value. **d** Calculated decay tau values. Axonal mitochondria exhibited significantly faster Ca^2+^ decay than dendritic mitochondria (axon *n* = 31 mitochondria from 31 axons, dendrite *n* = 16 mitochondria from 16 dendrites). **e** Schematic diagram of the stimulation protocol. Each stimulation consists of ten pulses at a frequency of 10 Hz. After the first stimulation, there is a ~ 7-min interval before the second stimulation. **f** Ca^2+^ responses after the first and second stimulations from axonal mitochondria. Representative Ca^2+^ traces and images from the control group (left) and cyclopiazonic acid (CPA)-treated group (right). **g** Both groups showed similar Ca^2+^ responses after the first and the second stimulations. No differences were observed in normalized peak ΔF/F_0_ values by the first peak value between the control and CPA-treated group (control *n* = 19 mitochondria from 19 axons, CPA *n* = 18 mitochondria from 18 axons). **h** Ca^2+^ responses after the first and the second stimulation from dendritic mitochondria. Representative Ca^2+^ traces and images from the control group (left) and CPA-treated group (right). **i** Control groups showed similar Ca^2+^ responses after the first and the second stimulations, whereas the CPA-treated group showed a significantly reduced Ca^2+^ response after the second stimulation. Ca^2+^ responses at the second stimulation in both groups were significantly different (control *n* = 15 mitochondria from 15 dendrites, CPA *n* = 17 mitochondria from 17 dendrites). Axonal and dendritic mitochondrial Ca^2+^ dynamics were monitored using mito-jGCaMP8m with mScarlet at 15–21 DIV. Unpaired *t* test was used for bar graphs. For Ca^2+^ imaging experiments, *n* indicates the number of analysed processes (one mitochondrion per axonal or dendritic process). Mitochondrial numbers are annotated at the bottom of bar graphs. Data are presented as mean ± SEM; bold lines indicate mean values, and shaded areas represent SEM. ***P < 0.001. n.s. not significant. Scale bar, 5 µm.

Recent advances in Ca^2+^ monitoring techniques have enabled the direct measurement of mitochondrial Ca^2+^ dynamics using mitochondria-targeted genetically encoded Ca^2+^ sensors^33^. We generated mitochondria-targeted-jGCaMP8m (mito-jGCaMP8m) by incorporating the mitochondria-targeting sequence of COXVIII and transfected it with mScarlet in cortical pyramidal neurons using ex utero electroporation at E15.5 (Supplementary Fig. 1a). The ex utero electroporation method selectively labels cortical pyramidal neurons by introducing plasmids to neural progenitors lining the lateral ventricles^34^. Following dissociated culture for 15–21 DIV, mitochondrial Ca^2+^ dynamics in dendrites and axons were monitored following stimulation of 10 action potentials (APs). In order to establish stable baselines during recording, while leaving evoked activity intact, we added low concentrations of TTX (less than 50 nM) to block spontaneous activity only (Supplementary Fig. 2a–e). To ensure comparability of mitochondrial Ca^2+^ dynamics within each compartment at similar Ca^2+^ levels, we locally applied electrical field potentials using a glass pipette containing an electrode near distal dendrites or axons (Supplementary Fig. 1b). We first assessed mitochondrial Ca^2+^ release properties between axons and dendrites post-stimulation (10 AP at 10 Hz). Surprisingly, following rapid Ca^2+^ uptake, axonal mitochondria exhibited a decay time constant twice as fast as that of dendritic mitochondria (20 s for axons, 40 s for dendrites. Figure 1a–d and Supplementary Video 1). We did not observe any significant correlation between the peak of mitochondrial matrix Ca^2+^ amplitude and the decay time under these conditions (Supplementary Fig. 1c). Because differences in baseline mitochondrial Ca^2+^ levels could potentially influence apparent decay kinetics, we also quantified pre-stimulus baseline fluorescence (F_0_). Dendritic mitochondria exhibited higher baseline fluorescence than axonal mitochondria (Supplementary Fig. 1d). However, regression analysis controlling for baseline F_0_ showed that the compartment effect on decay time constant (tau) remained significant, whereas F_0_ itself was not a significant predictor (Supplementary Fig. 1e). Consistent with this, model-estimated tau values at the mean baseline F_0_ remained higher in dendrites than in axons (Supplementary Fig. 1f). These findings suggest an intrinsic disparity in mitochondrial Ca^2+^ extrusion properties between axons and dendrites.

### Dendritic, but not axonal, mitochondrial Ca^2+^ uptake is largely dependent on ER-stored Ca^2+^ release in cortical pyramidal neurons

In dendrites, mitochondrial Ca^2+^ uptake relies heavily on Ca^2+^ released from the ER following synaptic stimulation^24^. However, in hippocampal neurons, unlike dendritic ER, axonal ER is capable of importing Ca^2+^ upon action potentials^35^. To compare mitochondrial Ca^2+^ uptake properties between axons and dendrites while minimizing the influence of ER, we added CPA, which inhibits the sarco/endoplasmic reticulum Ca^2+^-ATPase (SERCA) pump, thus depleting ER-stored Ca^2+^. As stimulation of distal dendrites was not sufficient to induce a substantial amount of ER Ca^2+^ release, the electrode was placed near the soma (Supplementary Fig. 3a), and this stimulation successfully induced ER Ca^2+^ release (Supplementary Fig. 3b). The ER store was fully refilled by 7 min after stimulation, whereas it remained depleted under CPA-treated conditions (Supplementary Fig. 3c). Based on these results, we applied two stimulus trains (10 APs at 10 Hz) separated by a 7-min interval (Fig. 1e). The first stimulation served to deplete the ER Ca^2+^ store, whereas the second assessed the response after ER depletion. A low concentration of TTX was included throughout the experiment to suppress spontaneous activity without affecting evoked responses.

Axonal mitochondria of cortical pyramidal neurons were unaffected by the addition of CPA (Fig. 1f, g and Supplementary Video 2), whereas dendritic mitochondrial Ca^2+^ uptake was significantly reduced (~77%) under CPA-treated conditions in agreement with a previous report^24^, suggesting that dendritic mitochondria inherently possess ER Ca^2+^ release dependency for Ca^2+^ uptake (Fig. 1h, i and Supplementary Video 3). Notably, this compartment-specific difference in the second-to-first stimulation ratio was maintained across the measured F_0_ range, arguing against baseline fluorescence as the primary explanation for this difference (Supplementary Fig. 1g). As the cytosolic Ca^2+^ responses in dendrites remained similar in the first and the second stimulations under CPA-treated conditions, the marked reduction in mitochondrial Ca^2+^ uptake during the second stimulation is unlikely to be explained by differences in cytosolic Ca^2+^ levels (Supplementary Fig. 3d). This could be because most ER-released Ca^2+^ might be transferred to the mitochondria, or cytosolic Ca^2+^ from the ER upon stimulation could reduce the Ca^2+^ gradient across the plasma membrane, thereby affecting extracellular Ca^2+^ import.

To further characterize ER Ca^2+^ dynamics underlying these compartmental differences, we monitored ER Ca^2+^ signals during both electrical and pharmacological stimulation (Supplementary Fig. 3e–j). Upon electrical stimulation, which is more physiological, dendritic ER released Ca^2+^ whereas axonal ER instead imported Ca^2+^, consistent with its proposed role as a Ca^2+^ sink during neuronal activity^35^. In contrast, caffeine-induced activation triggered robust ER Ca^2+^ release in both compartments, confirming that axonal ER can function as a Ca^2+^ source when pharmacologically stimulated and is not depleted at baseline. These compartment-specific ER Ca^2+^ responses make it unlikely that ER Ca^2+^ release provides a common explanation for the distinct mitochondrial Ca^2+^ decay kinetics, particularly the faster decay observed in axons. At the same time, structural factors such as mitochondrial morphology may also contribute to compartment-specific Ca^2+^ kinetics. However, morphology alone is unlikely to explain the faster axonal decay observed here, as elongation of axonal mitochondria has previously been reported to accelerate rather than slow mitochondrial Ca^2+^ decay^1^. Overall, these results indicate that mitochondrial Ca^2+^ uptake is governed by distinct Ca^2+^ sources in dendrites and axons, with dendritic mitochondria largely dependent on ER-stored Ca^2+^ release, whereas axonal mitochondria can sustain Ca^2+^ uptake without ER Ca^2+^ release during action potential firing.

### Mitochondrial Ca^2+^ regulatory components exhibit differential expression between dendrites and axons of cortical neurons

**Fig. 2:**
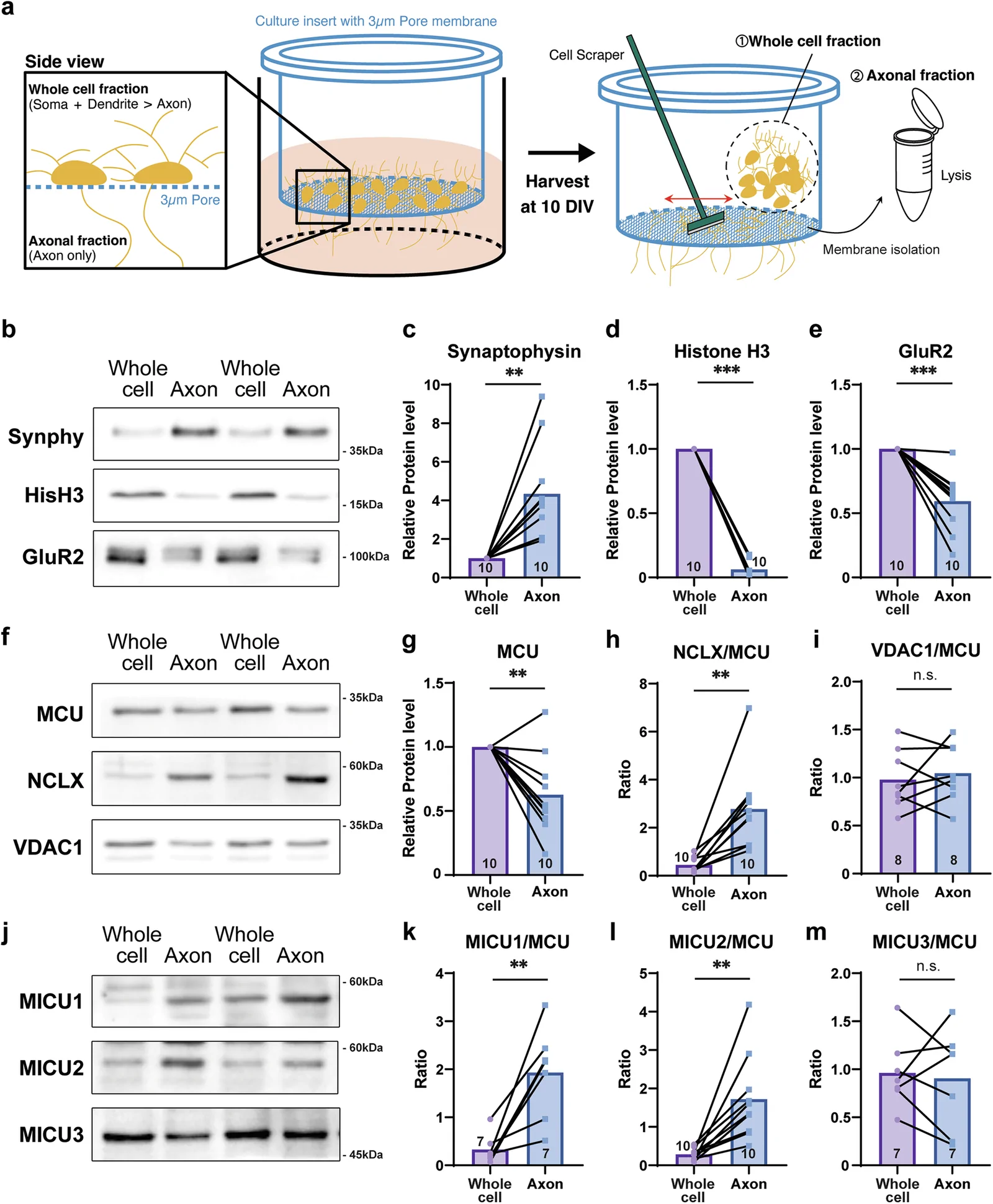
NCLX, MICU1 and MICU2 proteins are enriched in the axonal fraction. **a** Schematic representations of harvesting whole-cell fractions and axonal fractions separately. **b–e** Separation of whole-cell and axonal fractions is confirmed by synaptophysin (Synphy, presynapse/axon), histone H3 (HisH3, nuclear/cell body), and GluR2 (postsynapse/dendrite) levels. Synaptophysin level is significantly higher in the axonal fraction, but not histone H3 and GluR2. Representative Western blot images (**b**) and bar graph showing relative protein level of synaptophysin (**c**), histone H3 (**d**) and GluR2 (**e**). **f–m** Western blots of mitochondrial Ca^2+^ regulatory components in the whole-cell and axonal fractions. These protein levels were normalized by mitochondrial Ca^2+^ uniporter (MCU). Significantly higher ratios of NCLX, MICU1 and MICU2 to MCU are shown in the axon, whereas the ratios of VDAC1/MCU and MICU3/MCU are similar. Representative Western blot image (**f**, **j**) and bar graph showing the relative protein level of MCU (**g**) and the X:MCU ratio of NCLX (**h**), VDAC1 (**i**), MICU1 (**k**), MICU2 (**l**) and MICU3 (**m**). Relative protein levels were normalized to values from the whole cell fraction. Paired *t* test was used for bar graphs. Sample numbers are annotated at the bottom of bar graphs. Data are presented as mean ± SEM. **P < 0.01; ***P < 0.001. n.s. not significant.

To explore the molecular determinants underlying the functional differences in Ca^2+^ dynamics between axonal and dendritic mitochondria, cortical neurons were cultured on a 3 ${\rm{\mu}}{\rm{m}}$ porous membrane, allowing isolation of axons penetrating beneath the membrane (Fig. 2a). In addition, these cultures were incubated in the media without FBS to minimize the effect of astrocytes (Supplementary Fig. 4a). The whole neuron fraction above the membrane was collected using a cell scraper, followed by submersion of the membrane in the lysis buffer to extract the remaining axonal components. To validate the relative enrichment of the axonal fraction to the whole neuron fraction, the levels of histone H3 for the cell body, synaptophysin for the axon and GluR2 for the dendrite were examined. As anticipated, histone H3 and GluR2 levels were significantly lower in the axon fraction, whereas the presynaptic vesicle protein was enriched (Fig. 2b–e).

Subsequently, we investigated whether mitochondrial Ca^2+^ regulatory proteins differ between axonal and somatodendritic compartments. Given that axonal mitochondria are smaller in size and less dense than in the somatodendritic region^1,2^, the total mitochondrial protein content of the axon may be lower than that of dendrites when the same protein quantity is loaded. To test this, cortical pyramidal neurons expressing mito-YFP and HA-mCherry were immunostained with MCU antibody and intensities of MCU were similar in the dendritic and axonal mitochondria at 10 DIV (Supplementary Fig. 4b, c), even though the Western blot intensity is higher in the whole neuron fraction (Fig. 2f, g). Antibodies for other Ca^2+^ regulatory proteins are not available for immunocytochemistry; therefore, to mitigate this potential side effect, the protein X:MCU ratio was assessed.

Surprisingly, the mitochondrial Ca^2+^ release protein NCLX showed significantly higher expression in the axon, consistent with the faster decay times of axonal mitochondria (Fig. 2h). Regarding mitochondrial Ca^2+^ uptake, the ratio of MICUs to MCU is critical for the uptake capacity, with higher MICU1/2 to MCU increasing the opening threshold while cooperatively importing more Ca^2+^ than lower MICU1/2-associated MCU complexes^16,36–38^. VDAC1 is a non-selective channel passing ions, nucleotides and metabolites in the outer mitochondrial membrane, and MICU3 is known to be enriched in neurons enhancing MCU activity^35,39^. Interestingly, significantly higher ratios of MICU1/MCU and MICU2/MCU were observed in the axonal fraction, although VDAC1 and MICU3 to MCU ratios were similar between the whole-cell and the axon fractions (Fig. 2i–m). Similar to the MCU immunoblot, the non-normalized VDAC1, and MICU3 levels were lower in the axon owing to a lower mitochondrial amount as mentioned above, whereas NCLX, MICU1 and MICU2 amounts were still greater in the axon (Supplementary Fig. 4d–h).

To further ensure that the observed differences were not due to unequal mitochondrial abundance, we also compared TOM40 levels as an independent mitochondrial marker. Although TOM40 immunoblot intensity was lower in the axonal fraction, endogenous staining revealed comparable TOM40 expression in axonal and dendritic mitochondria (Supplementary Fig. 4i, j, k). Normalization of protein levels to TOM40 yielded results consistent with MCU-based normalization (Supplementary Fig. 4l–p), confirming that the observed differences are not attributable to uneven mitochondrial content. Finally, the expression of TMEM65, a known NCLX regulator, was comparable between compartments (Supplementary Fig. 4q–s). These results clearly show that NCLX, involved in mitochondrial Ca^2+^ release, and MICU1 and MICU2, related to mitochondrial Ca^2+^ uptake, are significantly enriched in axonal mitochondria.

### Neuronal compartment-specific mitochondrial Ca^2+^ dynamics and related protein expression profiles are conserved in hippocampal neurons

**Fig. 3:**
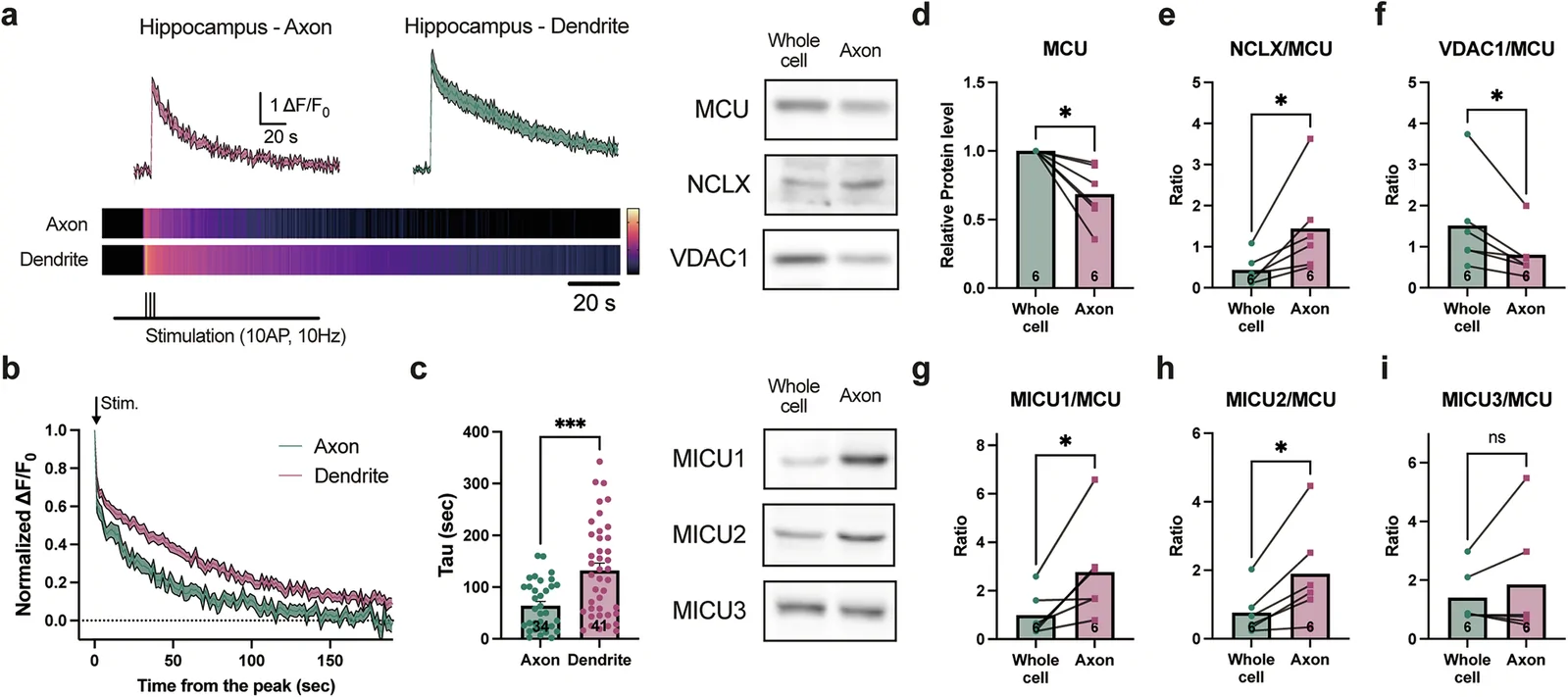
Mitochondrial Ca^2+^ dynamics and associated-protein expression patterns are mostly conserved in hippocampal neurons. Axonal mitochondria exhibited significantly faster Ca^2+^ decay than dendritic mitochondria. **a** Representative Ca^2+^ traces and corresponding line plots. **b** ΔF/F_0_ traces normalized to peak value. **c** Calculated decay tau values. (Axon *n* = 34 mitochondria from 34 axons, dendrite *n* = 41 mitochondria from 41 dendrites). **d–i** Western blots of mitochondrial Ca^2+^ regulatory components in the whole-cell and the axonal fractions. Protein levels were normalized with mitochondrial Ca^2+^ uniporter (MCU). NCLX, MICU1 and MICU2 showed significantly higher expression relative to MCU in the axonal fraction, whereas MICU3/MCU ratio was similar and VDAC1/MCU was slightly lower. Bar graph showing relative protein level of MCU (**d**) and X:MCU ratio of NCLX (**e**), VDAC1 (**f**), MICU1 (**g**), MICU2 (**h**) and MICU3 (**i**). Relative protein levels were normalized to values from the whole-cell fraction. Paired *t* test was used for bar graphs. For Ca^2+^ imaging experiments, *n* indicates the number of analysed processes (one mitochondrion per axonal or dendritic process). Mitochondrial numbers are annotated at the bottom of bar graphs. Sample numbers are annotated at the bottom of bar graphs. Data are presented as mean ± SEM; bold lines indicate mean values and shaded areas represent SEM. **P < 0.01; ***P < 0.001. n.s. not significant.

As the initial findings were based solely on cortical neurons, we next investigated whether the compartment-specific differences in mitochondrial Ca^2+^ regulation represent a generalizable feature of neuronal architecture or a neuron-type-dependent characteristic. To address this, we examined mitochondrial Ca^2+^ dynamics of hippocampal pyramidal neurons following ex utero hippocampal electroporation with mito-jGCaMP8m and mScarlet. Similar to cortical neurons, axonal mitochondria exhibited significantly faster Ca^2+^ decay than dendritic mitochondria, with average decay time constants of approximately 60 and 130 s, respectively (Fig. 3a–c). Moreover, Western blot analysis of isolated axons and whole-cell fractions revealed that mitochondrial Ca^2+^ extrusion protein NCLX, along with the uptake modulators MICU1 and MICU2, were enriched in the axons of hippocampal neurons, whereas proteins such as MICU3 were expressed at comparable levels across compartments and VDAC1 showed slightly lower levels in axons (Fig. 3d–i, Supplementary Fig. 5). Taken together, these findings indicate that the neuronal compartment-specific differences in mitochondrial Ca^2+^ dynamics and associated protein expression are conserved between cortical and hippocampal neurons.

### Axon-enriched MICU1 and MICU2 contribute to differential mitochondrial Ca^2+^ uptake properties between dendrites and axons

**Fig. 4:**
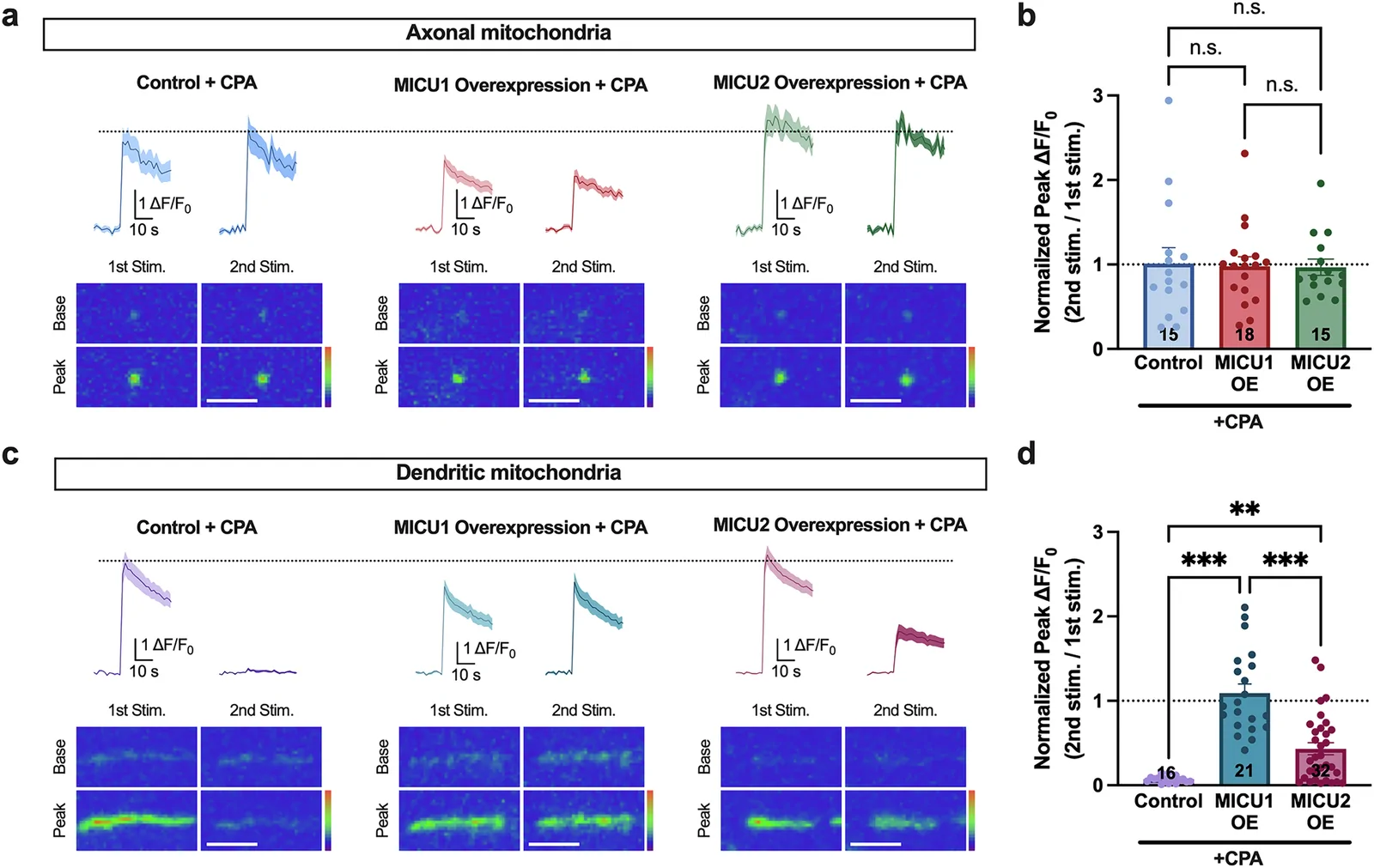
MICU1 or MICU2 overexpression is sufficient for ER-independent Ca^2+^ import of dendritic mitochondria. **a** Overexpression (OE) of MICU1 or MICU2 did not affect the Ca^2+^ uptake of axonal mitochondria. Representative Ca^2+^ traces and images of axonal mitochondria from the control group (left), the MICU1 OE group (middle) and the MICU2 OE group (right). **b** No differences were found in normalized peak ΔF/F_0_ values (peak from the second stimulation/peak from the first stimulation) between the three groups (control *n* = 15 mitochondria from 15 axons, MICU1 OE *n* = 18 mitochondria from 18 axons, MICU2 OE *n* = 15 mitochondria from 15 axons). **c** OE of MICU1 enabled dendritic mitochondria to sustain Ca^2+^ uptake under ER-depleted conditions, whereas OE of MICU2 showed a partial effect. Representative Ca^2+^ traces and images of dendritic mitochondria from the control group (left), the MICU1 OE group (middle) and the MICU2 OE group (right). **d** Normalized peak graph between the three groups under cyclopiazonic acid (CPA) treatment. Both MICU1 and MICU2 OE groups showed a significant increase in normalized peak value, with a larger effect in the MICU1 OE group than in the MICU2 OE group (control *n* = 16 mitochondria from 16 dendrites, MICU1 OE *n* = 21 mitochondria from 21 dendrites, MICU2 OE *n* = 32 mitochondria from 32 dendrites). Axonal and dendritic mitochondrial Ca^2+^ dynamics of control, MICU1-overexpressing or MICU2-overexpressing cortical pyramidal neurons were monitored using mito-jGCaMP8m at 15–21 DIV. All recordings were performed under 30-µM CPA treatment. One-way ANOVA with post hoc Tukey’s test was used for panels **b** and **d**. For Ca^2+^ imaging experiments, *n* indicates the number of analysed processes (one mitochondrion per axonal or dendritic process). Mitochondrial numbers are annotated at the bottom of the bar graphs. Data are presented as mean ± SEM; bold lines indicate mean values, and shaded areas represent SEM. **P < 0.01; ***P < 0.001. n.s. not significant. Scale bar, 5 µm.

Then, we checked whether the lower levels of MICU1 and MICU2 in dendrites contribute to distinct Ca^2+^ uptake properties compared with axonal mitochondria. To address this, we overexpressed MICU1 or MICU2 in cortical pyramidal neurons using ex utero electroporation, then measured mitochondrial Ca^2+^ dynamics with CPA treatment (Fig. 4, Supplementary Fig. 6a). Axonal mitochondria displayed no significant changes in the normalized peak level (second stimulation/first stimulation) upon MICU1 or MICU2 overexpression (Fig. 4a, b and Supplementary Video 4), although the absolute peak value was significantly lower in MICU1 overexpression and slightly higher in MICU2 overexpression (Fig. 4a and Supplementary Fig. 6b). Decreased mitochondrial Ca^2+^ level by MICU1 overexpression may be caused by tightening of the cristae junction, which could lead to reduction of cristae Ca^2+^ influx^40^. In contrast, dendritic mitochondrial Ca^2+^ uptake was dramatically increased upon the same stimulation condition (Fig. 4c, d, Supplementary Fig. 6c and Supplementary Video 5), suggesting that MICU1 or MICU2 overexpression enables dendritic mitochondria to take up Ca^2+^ even after ER stored Ca^2+^ depletion. As MICU2 interacts with MCU via MICU1 (refs.^37,38,41,42^), MICU2 overexpression alone has a weaker effect on altering Ca^2+^ regulatory function. Taken together, axonally enriched MICU1/2 grant ER independency, suggesting that MICU1/2 serve as key determinants for mitochondrial Ca^2+^ uptake modality.

### MICU1 overexpression increases the vulnerability of dendritic mitochondria to excitotoxic stress

A previous study showed that increased mitochondrial Ca^2+^ uptake by MCU overexpression exacerbates excitotoxic cell death owing to rapid loss of mitochondrial membrane potential following NMDA treatment^32^. ER-stored Ca^2+^ serves as the primary Ca^2+^ source for dendritic mitochondria, with resting ER Ca^2+^ levels reaching up to 1 mM^25,43^. Therefore, a massive influx of Ca^2+^ released from the ER and imported from the extracellular space, combined with elevated mitochondrial Ca^2+^ uptake due to MICU1 overexpression, could lead to rapid mitochondrial Ca^2+^ overload, potentially damaging them. To investigate this, we examined changes in dendritic mitochondrial membrane potential under excitotoxic conditions in neurons overexpressing MICU1. Mitochondrial membrane potential, represented as the F_mitochondria_/F_cytoplasm_, was monitored using TMRM. Under baseline conditions without NMDA stimulation, TMRM signals remained stable, whereas NMDA-induced excitotoxic conditions caused a TMRM intensity decline in both groups, with a markedly faster decrease observed in MICU1-overexpressing neurons (Supplementary Fig. 7a–c). F_mitochondria_/F_cytoplasm_ values were significantly lower at 1 and 5 min post-stimulation in MICU1-overexpressing dendritic mitochondria, with no difference observed before treatment (Supplementary Fig. 7d). Under control conditions with NMDA treatment, axonal and dendritic mitochondria exhibited comparable rates of TMRM signal decay, indicating similar membrane-potential stability and susceptibility to mitochondrial permeability transition (Supplementary Fig. 7e). To further determine whether MICU1 overexpression increases neuronal vulnerability under excitotoxic stress, we assessed cell viability after NMDA treatment. MICU1-overexpressing neurons displayed a significantly higher percentage of cell death than control neurons (Supplementary Fig. 7f, g). These findings indicate that MICU1 overexpression induces Ca^2+^ overload in dendritic mitochondria following NMDA treatment, increasing vulnerability to excitotoxicity. Consistent with these findings, excessive mitochondrial Ca^2+^ uptake by MICU1 overexpression further reduced neuronal survival. Based on these results, we conclude that low MICU1 expression in dendritic mitochondria limits the Ca^2+^ source to the ER, thus protecting mitochondria from excitotoxic stress.

### Faster axonal mitochondrial Ca^2+^ release is regulated by higher levels of NCLX compared with dendritic mitochondria

**Fig. 5:**
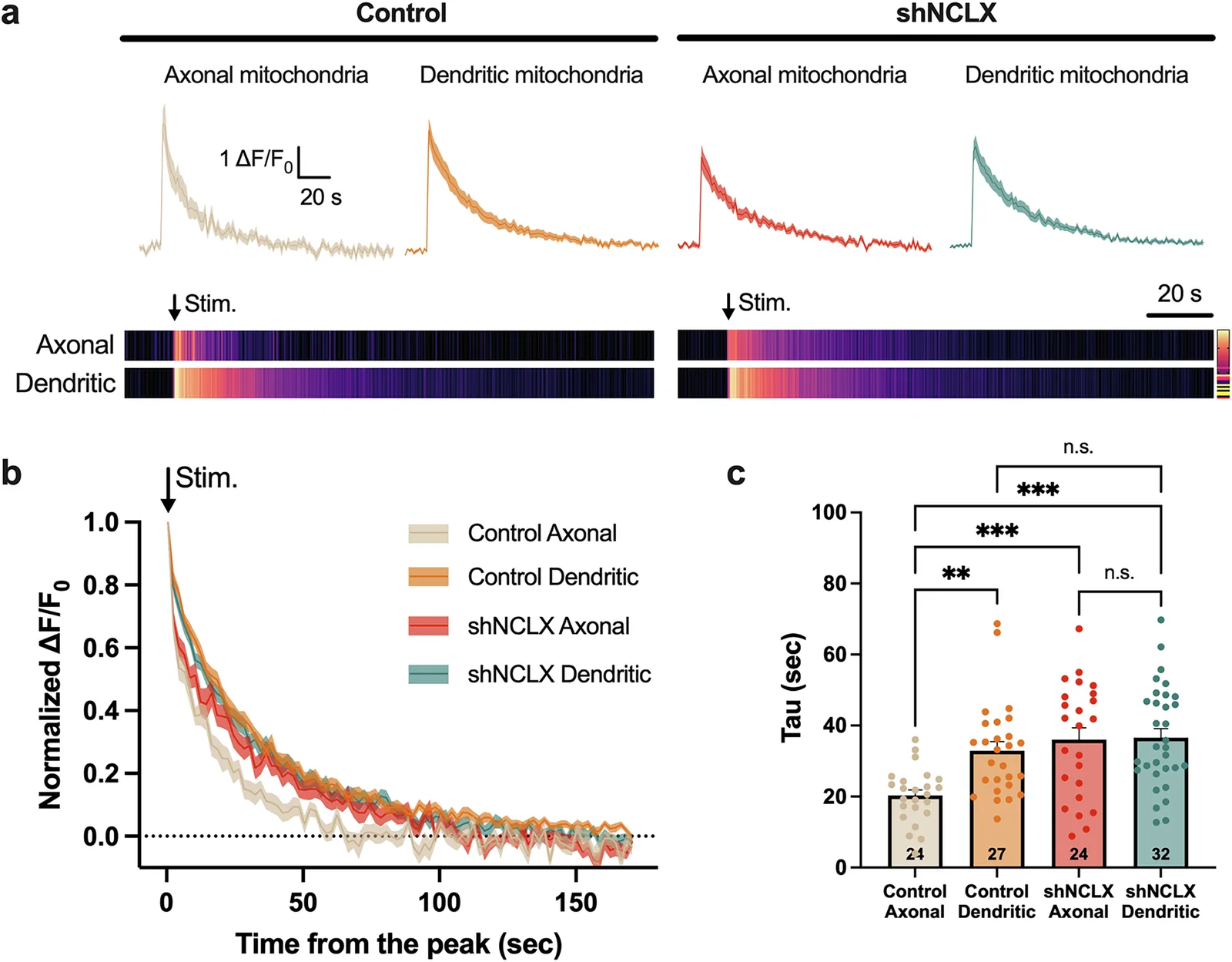
Faster Ca^2+^ efflux in axonal mitochondria attributed to elevated NCLX levels relative to dendritic mitochondria. **a** Representative Ca^2+^ traces of the control group (left) and short-hairpin NCLX (shNCLX) (NCLX knockdown) group (right). **b** Normalized ΔF/F_0_ traces by peak value. NCLX knockdown affects only the Ca^2+^ release of axonal mitochondria. **c** Calculated decay tau values. NCLX knockdown significantly increases decay tau of axonal mitochondria, but not in dendritic mitochondria (control axon *n* = 24 mitochondria from 24 axons, control dendrite *n* = 27 mitochondria from 27 dendrites, shNCLX axon *n* = 24 mitochondria from 24 axons, shNCLX dendrite *n* = 32 mitochondria from 32 dendrites). Axonal and dendritic mitochondrial Ca^2+^ dynamics of control or NCLX knockdown cortical pyramidal neurons were monitored using mito-jGCaMP8m at 15–21 DIV. One-way analysis of variance with post hoc Tukey’s test was used for panel **c**. For Ca^2+^ imaging experiments, *n* indicates the number of analysed processes (one mitochondrion per axonal or dendritic process). Mitochondrial numbers are annotated at the bottom of bar graphs. Data are presented as mean ± SEM; bold lines indicate mean values and shaded areas represent SEM. **P < 0.01; ***P < 0.001. n.s. not significant.

To investigate whether the elevated amount of NCLX in the axon contributes to its faster Ca^2+^ release property, we introduced a shNCLX plasmid co-expressing mTagBFP2, along with mito-jGCaMP8m and mScarlet as a cell filler (Supplementary Fig. 8). We then compared the axonal mitochondrial Ca^2+^ decay constant with that of dendrites following 10 AP stimulation. Under NCLX-deficient conditions, axonal mitochondria exhibited an extended Ca^2+^ release time, reaching a level similar to that of dendritic mitochondria (Fig. 5 and Supplementary Videos 6, 7). Interestingly, dendritic mitochondria showed no significant changes in the partial knockdown condition (Fig. 5 and Supplementary Fig. 8a); however, it appears to have a more pronounced impact on axonal mitochondrial function (Fig. 5). These results suggest that the rapid extrusion of axonal mitochondrial Ca^2+^ is derived from the higher levels of NCLX.

### NCLX knockdown significantly impairs axon development but not dendritic arborization

**Fig. 6:**
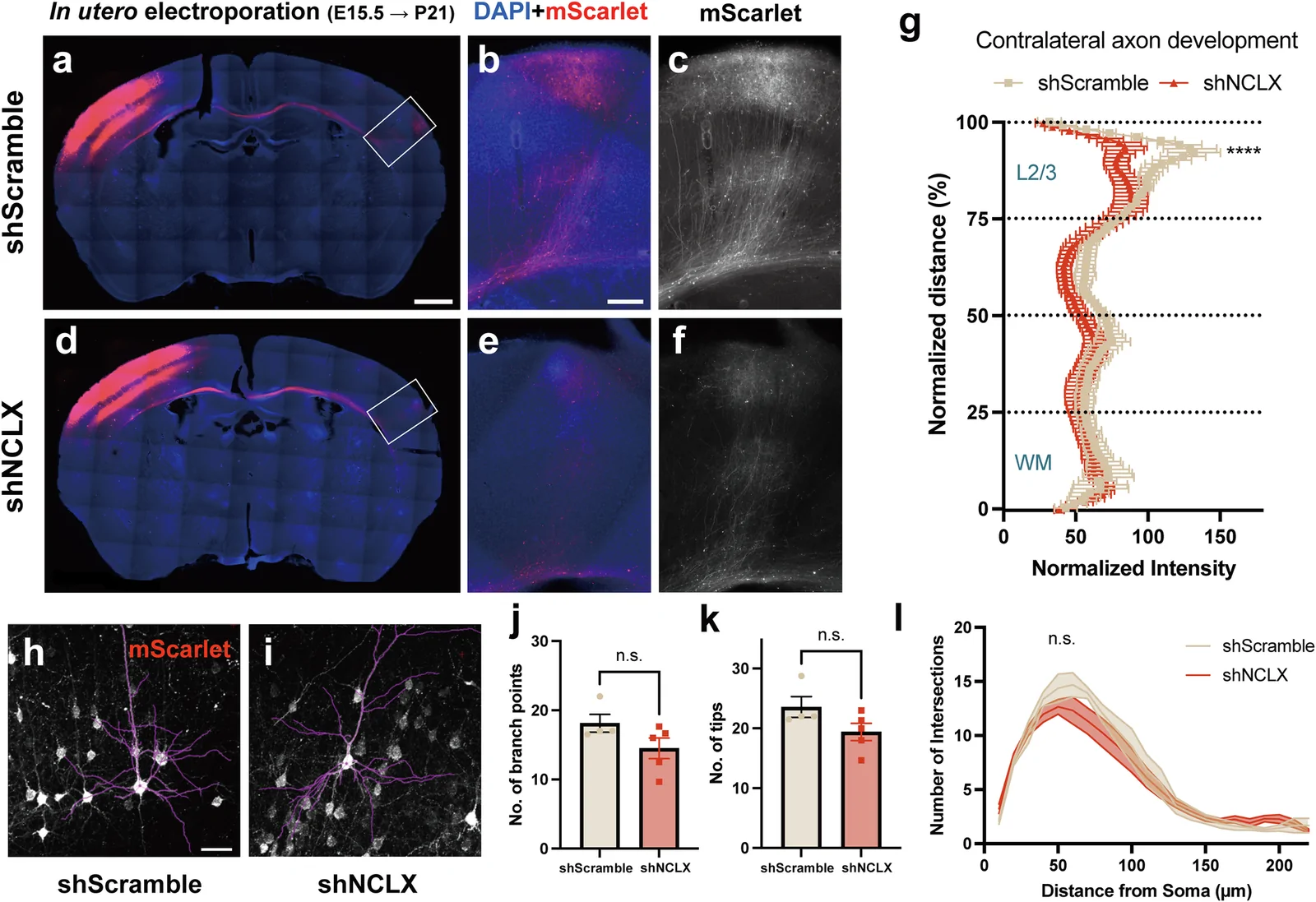
NCLX knockdown impairs axonal development but does not significantly affect dendritic branching. **a**, **d** Representative image of a coronal section of P21 mouse brain following cortical in utero electroporation with mScarlet and either with control short-hairpin RNA or short-hairpin NCLX (shNCLX). Scale bar = 1 mm. **b**, **c**, **e**, **f** High magnification of the box in panels **a** and **d**. Scale bar = 200 μm. **g** Quantification of mScarlet fluorescence along the radial axis of the contralateral cortex reveals decreased contralateral callosal axon projection signal upon NCLX knockdown. **h**, **i** Representative image of traced cortical neurons following in utero electroporation, a powerful technique used to introduce DNA plasmids into the developing central with mScarlet and either with control short-hairpin RNA or shNCLX. Scale bar = 40μm. **j–l** Quantification of dendritic branching using Sholl analysis. Parameters analysed include the number of branch points (**j**), the number of tips (**k**) and the number of intersections (**l**). Control *n* = 4 mice, shNCLX *n* = 5 mice. Data are presented as mean ± SEM; bold lines indicate mean values, and shaded areas represent SEM. Two-way analysis of variance with mixed-effects analysis with post hoc Sidak’s test was used for panels **g** and **l**, unpaired *t* test was used for panels **j** and **k**. ***P < 0.001. n.s. not significant.

Mutation or reduced expression of NCLX has been linked to mental retardation and neurodegenerative conditions including Alzheimer’s disease^44,45^. In particular, NCLX P367S mutation near the catalytic domain, which was identified in patients with severe intellectual disability, showed mitochondrial Ca^2+^ dynamics similar to those observed under NCLX KO conditions^45^. Therefore, we tested whether NCLX deficiency can affect neuronal development in a compartment-dependent manner. To this end, we performed unilateral in utero electroporation at E15.5 to introduce scrambled shRNA (shScramble) or shNCLX together with an mScarlet as a neuronal filler into layer 2/3 cortical pyramidal neurons and assessed their morphologies at P21. In control brains, callosal axons extended across the midline and formed a dense and stereotyped terminal projection pattern in the contralateral side, targeting both layers 2/3 and layer 5 (Fig. 6a–c). In contrast, NCLX knockdown led to a marked and layer-specific reduction in contralateral callosal axon projections, particularly in layers 2/3, despite normal trajectory and targeting (Fig. 6d–g). A complementary area-weighted intensity analysis across radial bins, which incorporates both fluorescence intensity and the covered area, was consistent with a reduction in upper-layer callosal axon signal in the shNCLX group (Supplementary Fig. 9a). To check the impact on dendrites, we reconstructed the dendritic morphologies from the ipsilateral part of the electroporated cortex. A mild reduction in complexity was noted in the shNCLX group, but the difference was not statistically significant (Fig. 6h–l), indicating that dendritic arborization is relatively resilient to NCLX deficiency. To further validate the axonal branching phenotype with a direct and quantitative approach, we analysed axonal morphology in cultured cortical neurons. Consistent with the in vivo results, NCLX knockdown significantly reduced the number of axonal branches without affecting total axon length (Supplementary Fig. 9b–f). Together, these findings suggest that the asymmetric expression of NCLX is associated with distinct developmental effects on axons and dendrites, with axonal development being more strongly affected by NCLX deficiency than dendritic development.

## Discussion

**Fig. 7:**
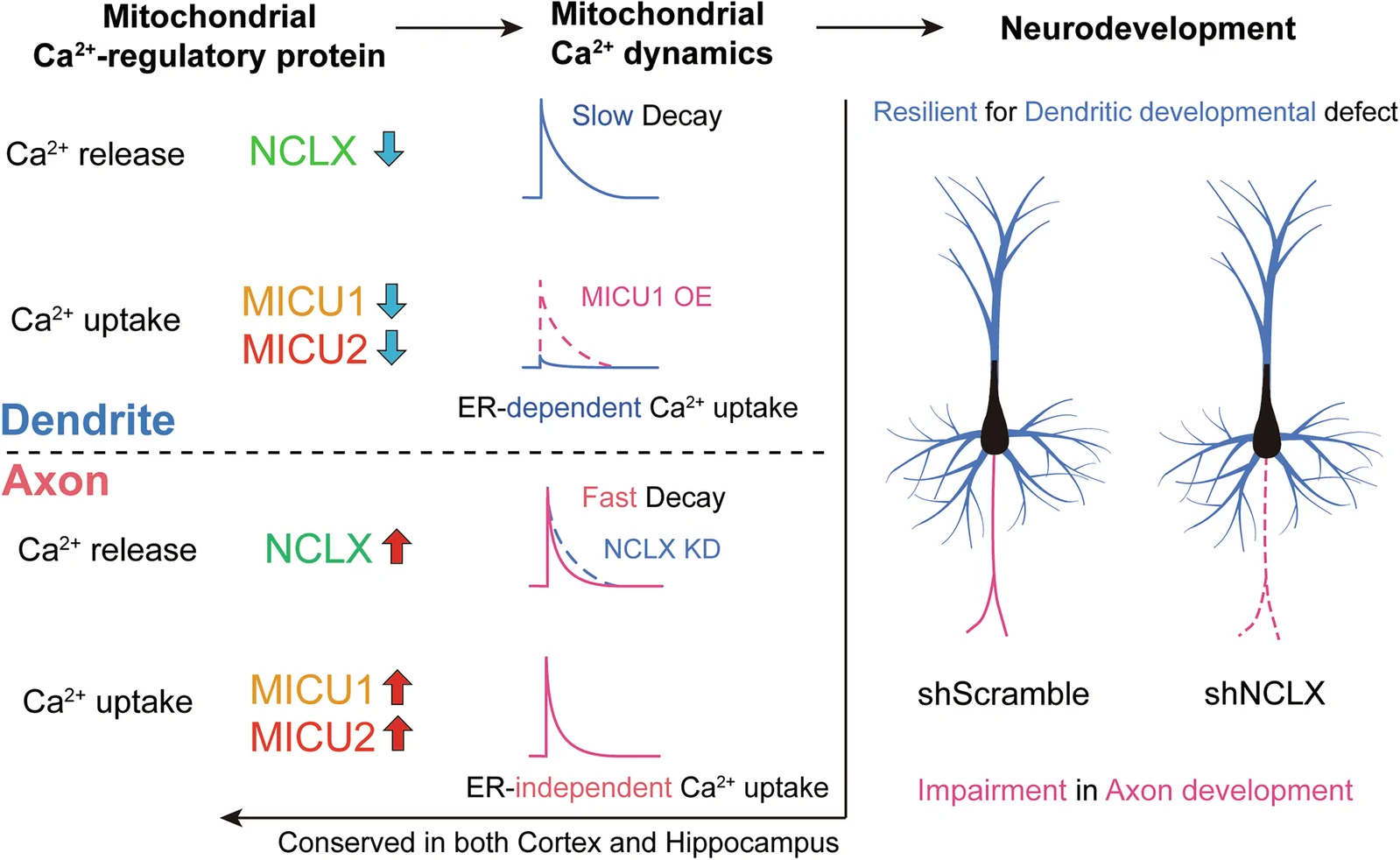
Compartment-specific asymmetry in mitochondrial Ca^2+^ regulation and functional properties. In dendrites (top), synaptic input promotes ER-stored Ca^2+^ transfer to mitochondria. In contrast to axonal mitochondria, lower MICU1 and MICU2 levels lead to ER-dependent mitochondrial Ca^2+^ uptake, with slower mitochondrial Ca^2+^ efflux owing to low NCLX expression. In axons (bottom), synaptic input drives both ER and mitochondrial Ca^2+^ uptake. High NCLX expression in the axon facilitates rapid mitochondrial Ca^2+^ clearance, whereas axon-enriched MICU1 and MICU2 support a more ER-independent mitochondrial Ca^2+^ uptake. NCLX knockdown slowed axonal mitochondrial Ca^2+^ release and was associated with impaired axon development, whereas dendritic development was relatively resilient.

The structural contrast of neuronal mitochondria found in dendrites and axons and their functional relevance have been studied most often independently. Here, we demonstrate striking differences in the Ca^2+^ regulation by dendritic and axonal mitochondria in cortical pyramidal neurons. Axonal mitochondria maintain uptake even when ER-derived Ca^2+^ is depleted and display faster Ca^2+^ release, in contrast to dendritic mitochondria, which show strong ER dependence and slower Ca^2+^ release. These differences are consistent with distinct mitochondrial Ca^2+^ regulatory molecular compositions within each compartment, with axons showing higher levels of NCLX, MICU1 and MICU2. Importantly, this asymmetry is conserved in hippocampal pyramidal neurons, indicating that this compartmental difference is a generalizable feature of excitatory neurons. MICU1 or MICU2 overexpression enhanced Ca^2+^ uptake only in dendrites, whereas NCLX knockdown selectively slowed mitochondrial Ca^2+^ release in axons. Finally, NCLX deficiency significantly impaired axon development both in vivo and in vitro, whereas dendritic branching was not significantly affected. In contrast, MICU1 overexpression significantly increased dendritic mitochondrial vulnerability upon excitotoxicity, revealing distinct developmental and stress-related phenotypes associated with compartment-specific mitochondrial Ca^2+^ regulation (Fig. 7). Taken together, our study is centred on defining compartment-specific mitochondrial functions and identifying the protein-level asymmetry underlying these functional differences.

A previous study demonstrates that overexpression of mental retardation-associated NCLX mutant mimics the mitochondrial Ca^2+^ dynamics observed in NCLX KO hippocampal neurons, causing delayed Ca^2+^ release from the mitochondrial matrix^45^. In addition, both presynaptic cytosolic Ca^2+^ concentration and neurotransmitter release are reduced in these KO neurons. Our NCLX-knockdown axonal mitochondria display prolonged matrix Ca^2+^ decay, which may lead to similar deficits in presynaptic release. In addition to the role of mitochondria-derived ATP in supporting local translation of cytoskeletal proteins during axon branching^46^, our findings are consistent with the idea that precise mitochondrial Ca^2+^ regulation contributes to presynaptic signalling and may be relevant to the observed axonal developmental phenotype^1,45,47^. Such altered presynaptic function could, in turn, impair axon development, a process known to be highly activity-dependent^47^. Nevertheless, the present data do not establish a direct causal link between prolonged axonal mitochondrial Ca^2+^ decay and the observed developmental phenotype. In addition, we acknowledge that fluorescence intensity-based quantification of callosal projections is an indirect measure and does not directly resolve single-axon branching architecture. Future studies employing sparse labelling, single-axon tracing, branch-point counting or 3D reconstruction approaches would provide a more direct assessment of axonal branching architecture.

Loss-of-function mutations in *MICU1* are associated with abnormalities in brain development, learning difficulties and movement disorders^48–50^. Similarly, null mutation in *MICU2* has also been found in patients having neurodevelopmental disorders with severe cognitive impairment and spasticity^51^. However, the progression of cellular defects by these mutations, particularly at the neuronal level, remains unclear. Defective mitochondrial Ca^2+^ uptake can elevate presynaptic Ca^2+^ and alter release properties, which can affect axon branching^4,30,52^. Therefore, as observed in NCLX-deficient neurons, MICU1- or MICU2-mutant neurons may exhibit more significant axonal than dendritic abnormalities.

Beyond neurodevelopment, mitochondrial Ca^2+^ dysregulation is also strongly related to neurodegenerative diseases^11,20,21^. A former investigation showed that Parkinson’s disease-derived LRRK2 mutant increased mitochondrial Ca^2+^ uptake via MCU and MICU1 upregulation, resulting in dendritic shortening^19^. In addition, restoring the mitochondrial Ca^2+^ homeostasis by overexpressing the NCLX active form rescued dendritic length, highlighting the importance of balanced mitochondrial Ca^2+^ dynamics for both axonal and dendritic arborization. Therefore, the compartment-specific enrichment of mitochondrial Ca^2+^ regulators may exert asymmetric effects on each compartment.

MICU1 plays a dual role in regulating mitochondrial Ca^2+^ uptake, preventing excessive Ca^2+^ entry at low cytosolic Ca^2+^ concentrations while enhancing Ca^2+^ uptake at higher concentrations^36,53^. Interestingly, both silencing and overexpression of MICU1 lead to mitochondrial Ca^2+^ overloading, which triggers cell death^37,54,55^. In our study, MICU1 overexpression enabled ER-independent Ca^2+^ uptake and led to rapid mitochondrial depolarization under excitotoxic conditions (Supplementary Fig. 7). Taken together, asymmetric distribution of MICU1 in neurons is critical, not only for defining the source of Ca^2+^ entry but also for protecting mitochondrial integrity and cell viability.

Based on previous studies, we propose additional functional implications of the distinct mitochondrial Ca^2+^ regulation observed in axonal and dendritic compartments:

(1) The distinct mitochondrial Ca^2+^ dynamics observed in axons and dendrites may have implications for compartment-specific energetic regulation. Synapses consume a substantial amount of ATP, with dendrites thought to account for a larger fraction of this demand than axons^56,57^. Given the established role of mitochondrial Ca^2+^ in metabolic regulation^11^, our findings may be consistent with distinct physiological requirements across neuronal compartments. In particular, the slower Ca^2+^ decay observed in dendritic mitochondria (Fig. 1) may be suited to supporting greater energetic demand, consistent with recent evidence that prolonged mitochondrial Ca^2+^ retention can enhance neuronal mitochondrial metabolism and ATP production^58^. ATP is also crucial for presynaptic function, with presynaptic mitochondria suggested to generate ATP in response to Ca^2+^ influx^35^. However, glycolysis can also provide energy at presynaptic sites in multiple species^59,60^. This process could respond faster upon action potential arrival than oxidative phosphorylation and support mitochondria-free presynapses, which constitute more than half of excitatory presynaptic boutons. Intriguingly, recent preprint studies suggest that axonal mitochondria contain less mitochondrial DNA^61,62^ and even consume the ATP rather than generating it, unlike dendritic mitochondria, possibly owing to complex V hydrolysis^61^.

(2) Compartment-specific mitochondrial Ca^2+^ dynamics may finely modulate synaptic communication. Mitochondrial Ca^2+^ buffering affects cytosolic Ca^2+^ concentration and this can contribute to Ca^2+^-activated K^+^ channel (K_Ca_) activity^63^. Interestingly, K_Ca_ channels are expressed in a compartment-specific manner^64^. The activity of large-conductance Ca^2+^-activated K^+^ channels (BK channels), which are enriched in axons, is critical for spike fidelity^65^. A rapid Ca^2+^ release from axonal mitochondria may lead to a cytosolic Ca^2+^ transient that enhances BK channel activity, thus supporting spike fidelity in the axon. In contrast, some of the small-conductance Ca^2+^-activated K^+^ channels such as SK1 and SK2, are enriched in soma and dendrites^64^. Given that dendritic mitochondria release Ca^2+^ at slower rates, SK channels may be activated more slowly. This could prevent excessive hyperpolarization of the membrane potential during synaptic events such as burst firing, thereby maintaining sensitivity to subsequent synaptic events.

Although our study defines compartment-specific mitochondrial Ca^2+^ dynamics and identifies the protein-level asymmetry associated with these functional differences, how this protein asymmetry is established remains unresolved. Additionally, a more definitive understanding of the endogenous compartment-specific distribution of these regulators will require approaches that can assess their localization with higher spatial precision. We also cannot completely exclude the possibility that dendritic ER Ca^2+^ release and/or ER–mitochondria coupling contribute(s) to the slower mitochondrial Ca^2+^ decay observed in dendrites, because depletion of ER-stored Ca^2+^ itself suppresses dendritic mitochondrial Ca^2+^ uptake. In addition, structural factors such as ER–mitochondria contact site organization and local Ca^2+^ microdomain architecture may also contribute to the compartment-specific Ca^2+^ kinetics, which cannot be fully resolved by the imaging approaches employed in the present study. Therefore, future studies will be required to elucidate the mechanisms underlying the polarized distribution of mitochondrial components. Possible mechanisms include mRNA localization and local translation, post-transcriptional protein targeting and import, and selective trafficking or anchoring of mitochondria themselves^66^. In addition, another layer of regulation may involve spatial organization within the mitochondrial inner membrane, as previous studies have suggested that uptake and release components can occupy distinct submitochondrial regions^67^. More broadly, recent in vivo proteomic and translatomic work also supports the idea that intraneuronal mitochondrial composition is heterogeneous and context dependent^62^, underscoring the need for more precise mechanistic studies.

Overall, our study unveiled novel cellular mechanisms specifying neuronal polarized function via compartment-dependent mitochondrial Ca^2+^ regulation. These findings shine lights on the importance of mitochondrial molecular variability for physiological and pathophysiological studies.

## Supplementary information

## Data Availability

All data reported in this paper will be shared by the corresponding authors upon request. This paper does not report original code. Any additional information required to reanalyse the data reported in this work paper is available from the corresponding authors upon request.
