# Supplementary material for: Asymmetric distribution of mitochondrial Ca2+ regulators specifies compartment-specific mitochondrial function and neuronal development
Source: Exp Mol Med. 2026 Aug 5;58(8):2688–700. doi: 10.1038/s12276-026-01803-2 (PMC13538658; doi:10.1038/s12276-026-01803-2)
Supplement: Supplementary file 1 [file 12276_2026_1803_MOESM1_ESM.pdf]

Supplementary Figures

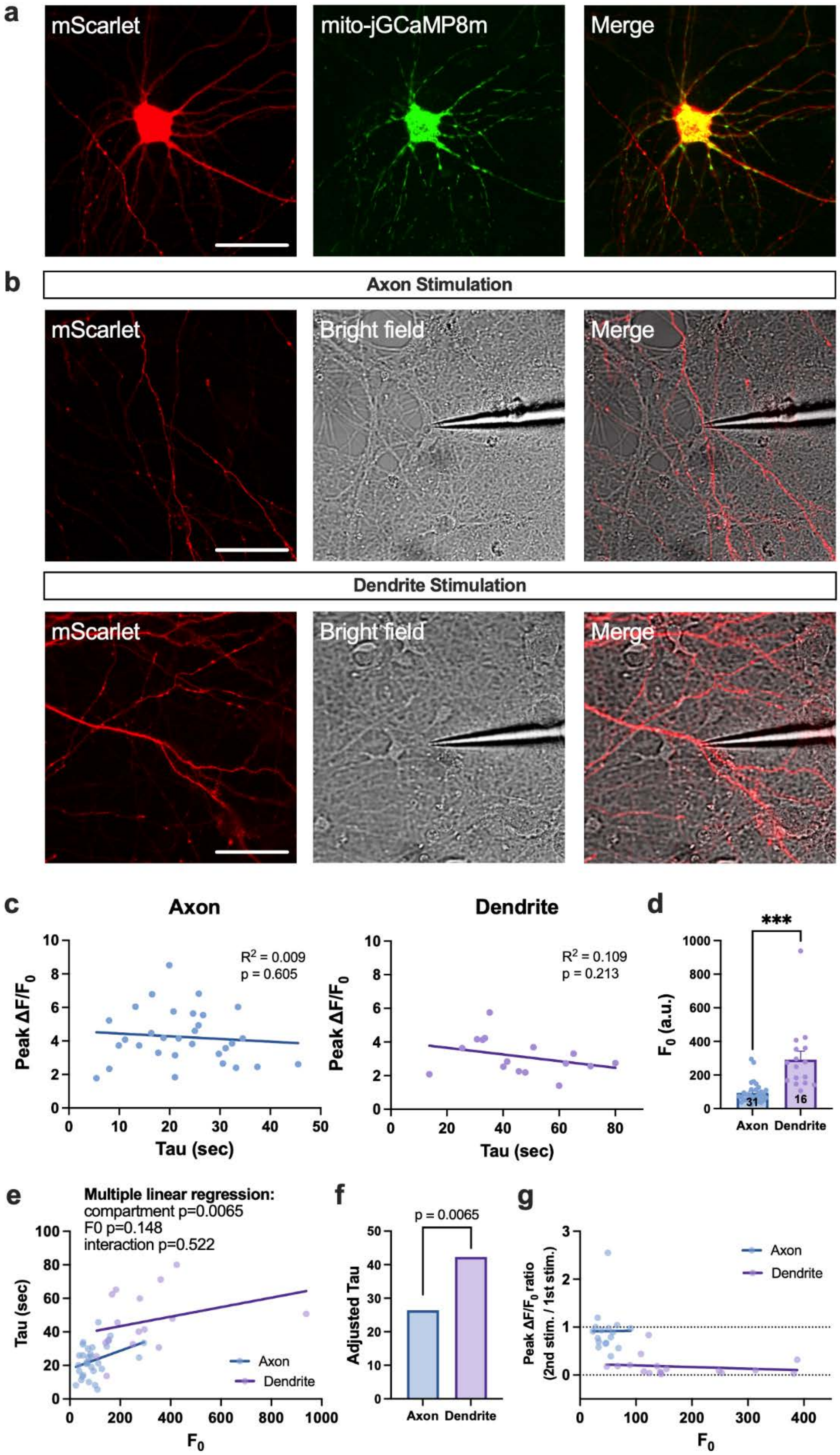

Supplementary Fig. 1. Representative images of recorded neurons and correlation between peak intensity and decay tau in mitochondria. (a) Representative images of recorded neurons. pCAG::mScarlet and pCAG::mito-jGCaMP8m were electroporated into the cortical pyramidal neurons and monitored at 17-21 DIV. Scale bar, 50 µm. (b) Representative images of axon (upper) and dendrite (lower) stimulation. The stimulation electrode (glass capillary) was placed <10 µm toward the z-axis from the target (middle, bright field). (c) Peak mitochondrial Ca^{2+} responses did not correlate with decay kinetics (τ) in either compartment. (d) Pre-stimulus baseline fluorescence (F_0) was higher in dendritic than in axonal mitochondria. (e) Multiple linear regression analysis showed that the compartment effect remained significant after controlling for F_0 (compartment $p = 0.0065$), whereas the main effect of F_0 ($p = 0.148$) and the interaction term ($p = 0.522$) were not significant. (f) Adjusted tau values represent model-estimated tau at the mean baseline F_0. (g) The compartment-specific difference in the 2nd stimulation/1st stimulation ratio was maintained across the measured F_0 range. Unpaired t-test was used for panel d. Sample numbers are indicated at the bottom of the bar graph. Data are presented as mean±SEM; bold lines indicate mean values, and shaded areas represent SEM. ***$p < 0.001$.

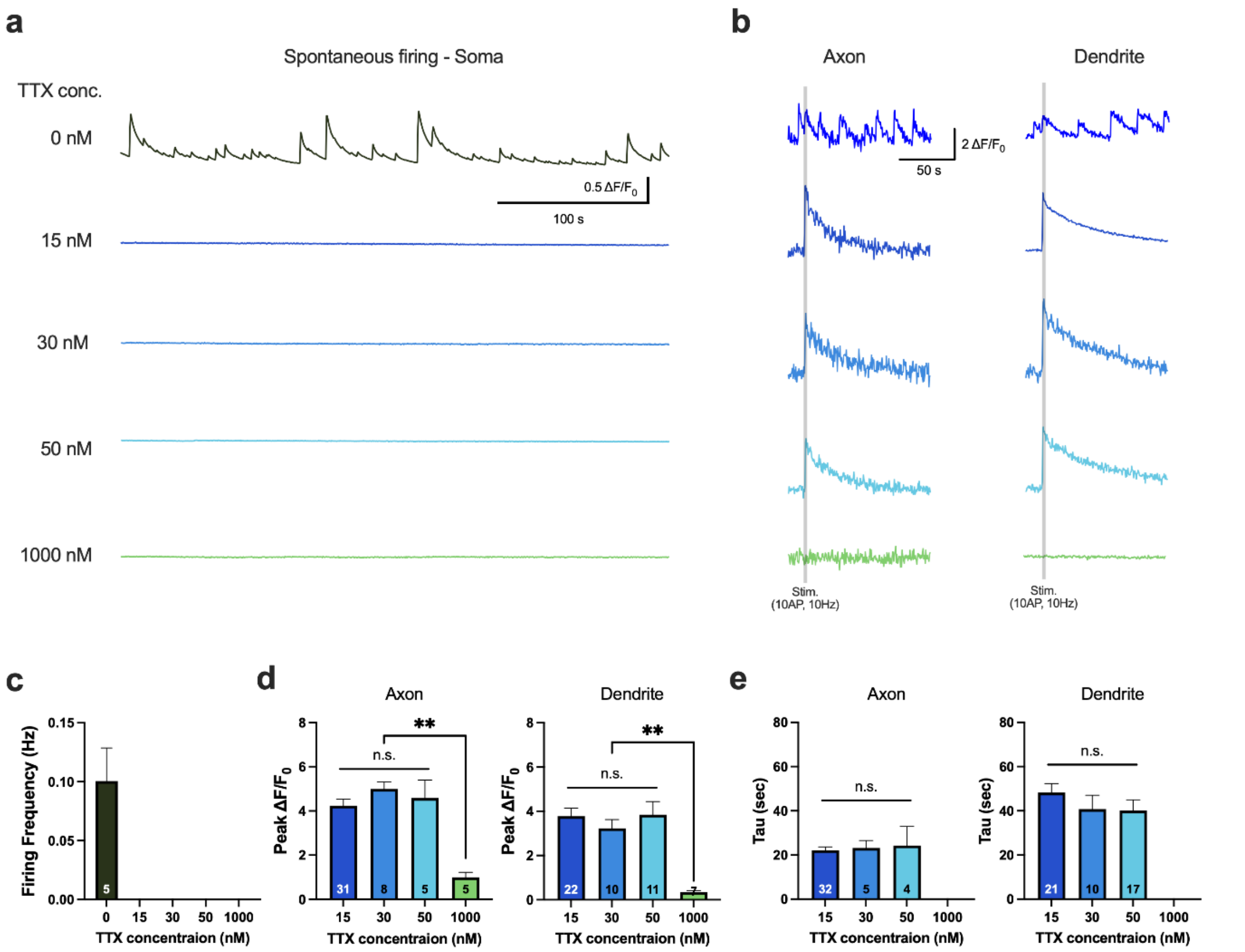

Supplementary Fig. 2. Mitochondrial Ca^{2+} transients under various tetrodotoxin (TTX) concentrations. (a, c) Spontaneous firing was observed with no TTX treatment (0 nM), but 15 nM or higher concentrations of TTX blocked the spontaneous firing. (b, d) Evoked Ca^{2+} responses in axonal and dendritic mitochondria were similar between 15 nM and 50 nM TTX. The Ca^{2+} response was relatively smaller without TTX treatment, and almost no response was observed at 1000 nM TTX. (e) TTX treatment did not affect mitochondrial Ca^{2+} decay tau. One-way ANOVA with *post hoc* Tukey's test was used for panels d and e. Mitochondrial numbers are annotated at the bottom of the bar graphs (one mitochondrion was analyzed per axonal or dendritic process). Somatic, Axonal and dendritic mitochondrial Ca^{2+} dynamics of cortical pyramidal neurons were monitored using mito-jGCaMP8m at 15-21 DIV. Data are presented as mean±SEM. $^{**}p < 0.01$. $^{***}p < 0.001$. n.s., not significant.

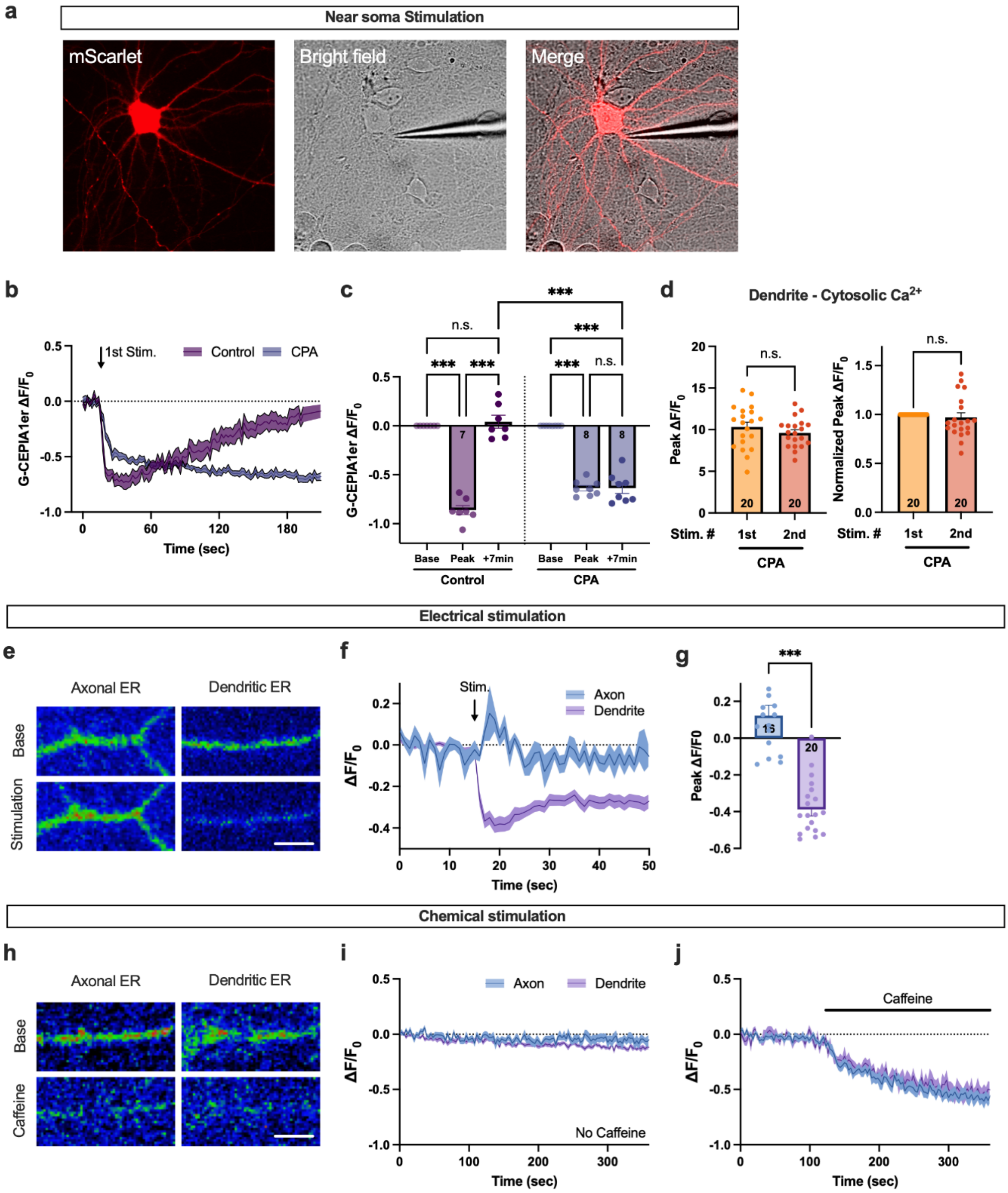

Supplementary Fig. 3. Stimulation protocol and representative images of recorded neurons. Axonal and dendritic mitochondrial Ca^{2+} dynamics of CPA-treated cortical pyramidal neurons were monitored using mito-jGCaMP8m at 15-21 DIV. (a) A representative image of the stimulation method. The stimulation electrode (glass capillary) was placed <10 µm toward the z-axis from the target (middle, bright field). Scale bar, 50 µm. (b, c) Dendritic ER Ca^{2+} dynamics was monitored by G-CEPIA1er with CPA treatment. (b) The control group showed a rapid signal reduction after the first stimulation, but the reduced signal almost recovered around 3 min after stimulation (n=7). In contrast, the CPA group showed a sustained reduction of the signal after the first stimulation (n=8). (c) Quantification of ER

Ca^{2+} signals at baseline, peak, and 7-min after the first stimulation. The signal from the control group was significantly reduced at peak and fully recovered within 7-min after stimulation. In contrast, the reduced signal remained largely unchanged until 7-min after stimulation in the CPA-treated group. At 7-min after stimulation, the fluorescence signal between the control and CPA group was significantly different. (d) Cytosolic Ca^{2+} levels from the dendrite were not different between the first and the second stimulation under CPA-treated condition. (e–g) ER Ca^{2+} dynamics during electrical stimulation. ER Ca^{2+} signals were recorded using ER-GCaMP6f-150 in dendritic and axonal compartments during electrical stimulation (10 pulses at 10 Hz). (e) Representative traces of ER Ca^{2+} responses. (f) Averaged $\Delta F/F_0$ traces showing that dendritic ER released Ca^{2+} immediately after stimulation, whereas axonal ER accumulated Ca^{2+} under the same condition (Axon n=15 axonal processes, Dendrite n=20 dendritic processes). Scale bar, 10 µm. (g) Quantification of ER Ca^{2+} changes at baseline and peak. Dendritic ER showed a significant decrease in fluorescence intensity after stimulation, while axonal ER showed an increase. (h–j) ER Ca^{2+} dynamics during caffeine-induced stimulation. (h) Representative fluorescence traces showing ER Ca^{2+} release in response to local perfusion of 100 mM caffeine. Scale bar, 10 µm. (i) Averaged $\Delta F/F_0$ traces of both dendritic and axonal ER before caffeine application (Axon n=16 axonal processes, Dendrite n=20 dendritic processes). (j) Averaged $\Delta F/F_0$ traces of both dendritic and axonal ER under caffeine application (Axon n=10 axonal processes, Dendrite n=13 dendritic processes). Both dendritic and axonal ER showed significant Ca^{2+} release. One-way ANOVA with *post hoc* Sidak's test was used for panel c. Unpaired t-test was used for panel d. Sample numbers are indicated at the bottom of the bar graph. Data are presented as mean±SEM; bold lines indicate mean values, and shaded areas represent SEM. ***$p < 0.001$. n.s., not significant.

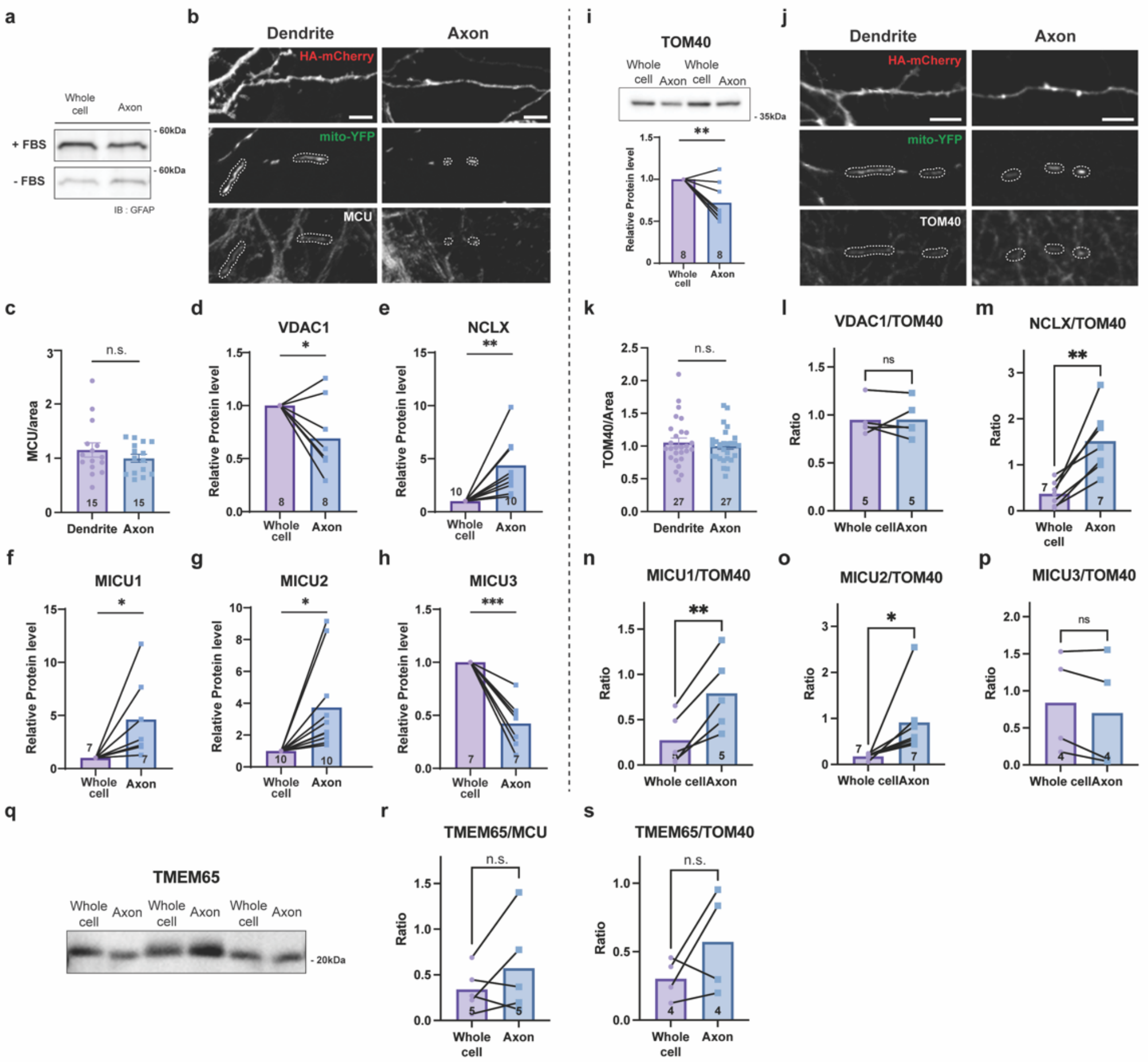

Supplementary Fig. 4. Relative expression levels of mitochondrial Ca^{2+} regulatory proteins.

(a) Culture medium without FBS showed lower levels of GFAP (astrocyte marker) in both axonal and whole-cell fraction at 10 DIV. (b, c) Endogenous MCU levels in dendritic and axonal mitochondria of cortical pyramidal neurons were quantified using immunostaining at 10 DIV. MCU expression in dendritic and axonal mitochondria is similar. (d–h) Western blot results of mitochondrial Ca^{2+} regulatory protein levels without MCU normalization. VDAC1 (d), NCLX (e), MICU1 (f), MICU2 (g), and MICU3 (h). (i) Western blot result of TOM40. (j, k) Endogenous TOM40 levels in dendritic and axonal mitochondria of cortical pyramidal neurons were quantified using immunostaining at 10 DIV. TOM40 expression in dendritic and axonal mitochondria is similar. (l–p) X:TOM40 ratio of VDAC1 (l), NCLX (m), MICU1 (n), MICU2 (o), and MICU3 (p). (q–s) TMEM65 Western blot results. TMEM65:MCU ratio (r) and TMEM65:TOM40 ratio (s). Relative protein levels were normalized to values from the whole-cell fraction. Paired t-test was used for panels d to i and l to s. Unpaired t-test was used for panels c and k. Sample numbers are annotated at the bottom of bar graphs. Data are presented as mean±SEM. $*p < 0.05$, $**p < 0.01$, $***p < 0.001$. n.s., not significant. Scale bar, 10 µm.

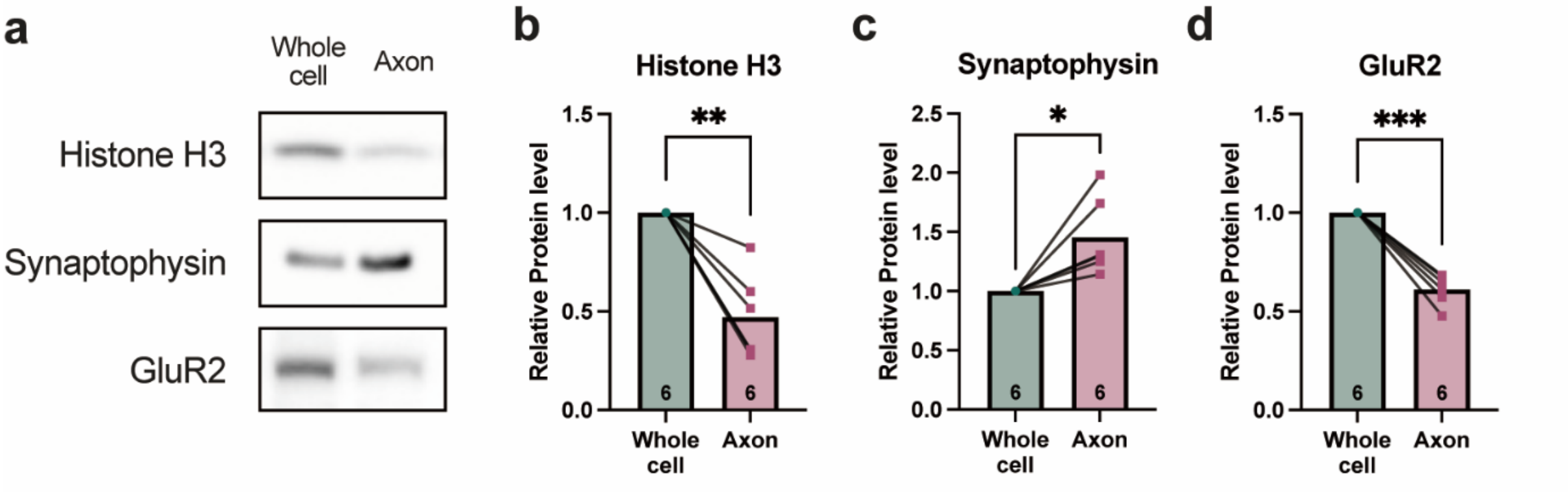

Supplementary Fig. 5. Validation of axon isolation in primary hippocampal culture. Separation of whole cell and axonal fraction is confirmed by synaptophysin (presynapse/axon), histone H3 (nuclear/cell body), and GluR2 (postsynapse/dendrite) levels. (a) Representative Western blot images. Bar graph showing synaptophysin (c) level is significantly higher in the axonal fraction, but not histone H3 (b) and GluR2 (d). Relative protein levels were normalized to values from the whole cell fraction. Paired t-test. Sample numbers are annotated at the bottom of bar graphs. Data are presented as mean±SEM. *$p < 0.05$. **$p < 0.01$. ***$p < 0.001$.

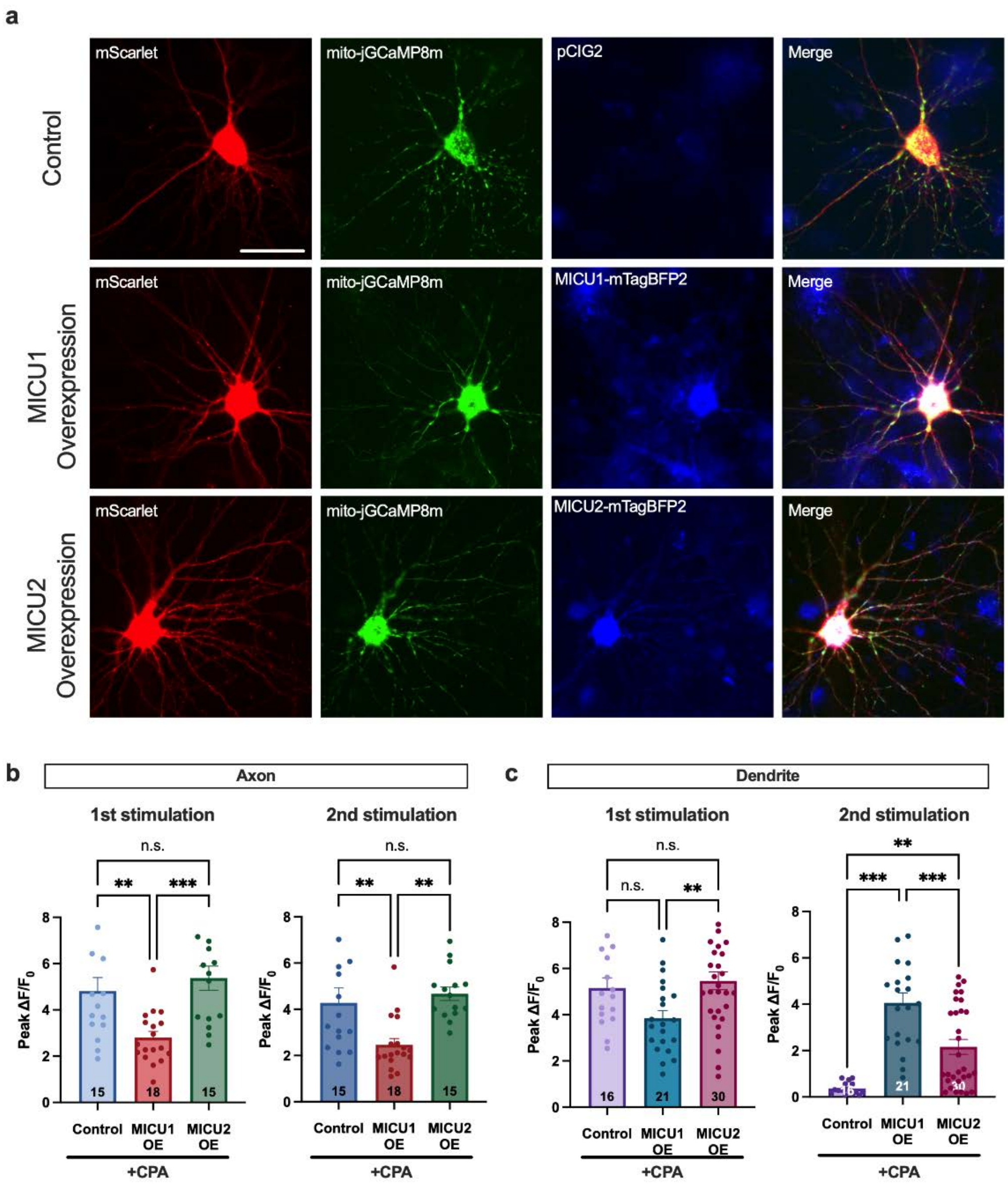

Supplementary Fig. 6. Validation of MICU1 and MICU2 overexpression and representative images of recorded neurons. (a) Representative images of recorded neurons. pCAG::mScarlet, pCAG::mito-jGCaMP8m and pCIG2 or pCAG::MICU1-mTagBFP2 or pCAG::MICU2-mTagBFP2 were electroporated into the cortical pyramidal neuron. (b, c) The peak intensity from axonal and dendritic mitochondria. (b) In axonal mitochondria, peak intensity was reduced and increased in the MICU1 and MICU2 overexpression groups, respectively. In the MICU1 overexpression group, the significant reduction was shown at the 2nd stimulation, but in the MICU2 overexpression group, there was a tendency of increased Ca^{2+} uptake at the 1st stimulation (c) In dendritic mitochondria, both MICU1 and MICU2 overexpression enhanced Ca^{2+} uptake at the 2nd stimulation, but in different amounts. Compared to the control group, the MICU1 overexpression group showed considerably higher peak intensity after ER-Ca^{2+} depletion, but partial enhancement by MICU2 overexpression was observed. One-way ANOVA with post hoc Tukey's test was used for panels c, d. Sample numbers are annotated at the bottom of bar graphs. Scale bar, 50 µm.

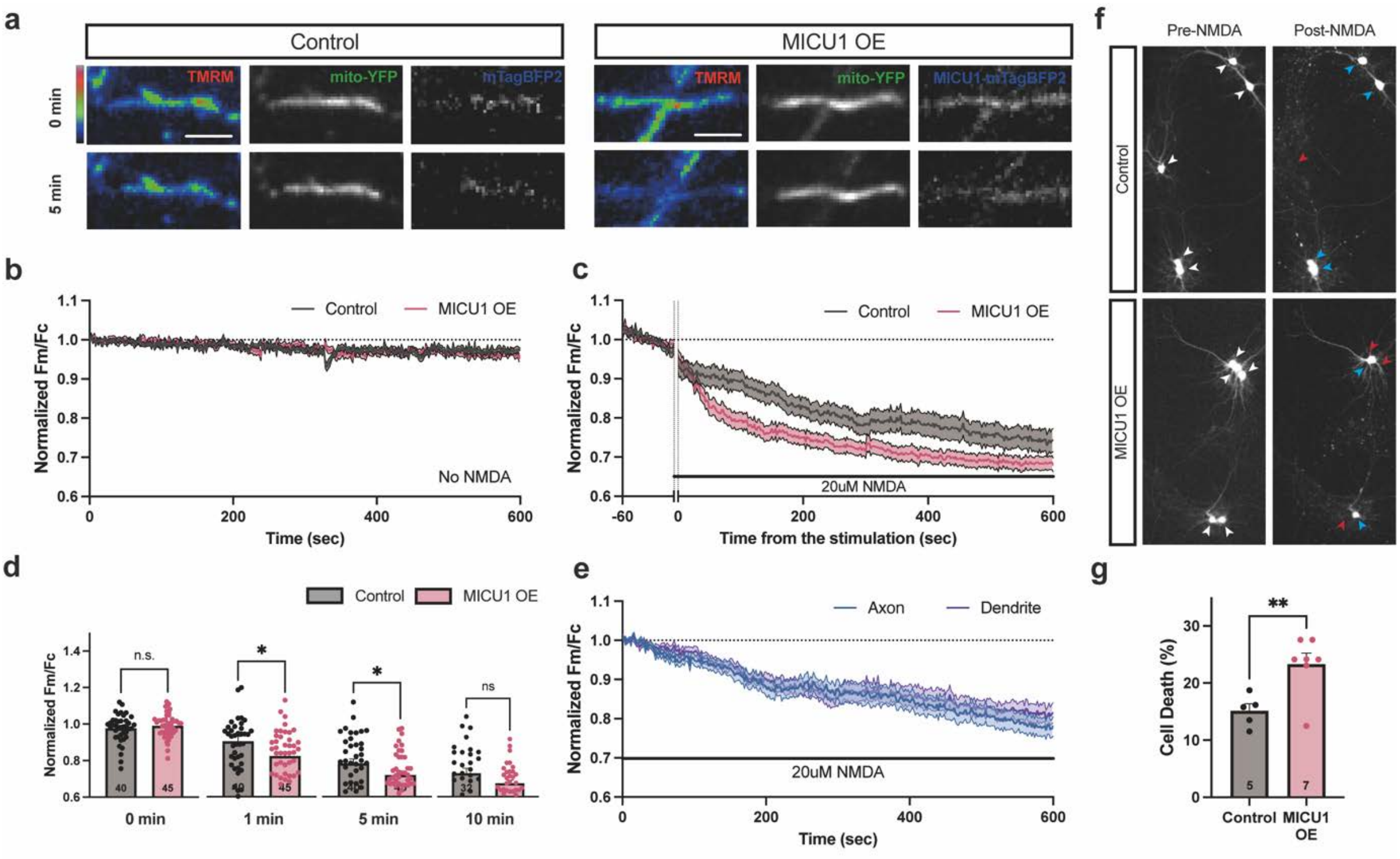

Supplementary Fig. 7. MICU1 overexpression accelerates the decrease in mitochondrial membrane potential and reduces cell viability under excitotoxic conditions. (a) Representative images of control and MICU1 overexpression (MICU1 OE) neurons expressing TMRM for mitochondrial membrane potential measurement. (b) Representative traces of mitochondrial membrane potential ($F_{mitochondria}/F_{cytoplasm}$, Fm/Fc) under baseline (no NMDA) conditions showing no difference between control and MICU1 OE (control n=43, MICU1 OE n=48). (c) Time course of Fm/Fc changes in response to 20 µM NMDA stimulation in control and MICU1 OE neurons. (d) Quantification of Fm/Fc at 0, 1, 5, and 10 minutes after NMDA stimulation (40 mitochondria from 40 dendrites for control, 45 mitochondria from 45 dendrites for MICU1 OE). Fm/Fc values were significantly lower in MICU1 OE at 1 and 5 minutes but not at 0 or 10 minutes. (e) Comparison of axonal and dendritic mitochondrial Fm/Fc changes under excitotoxic conditions in control neurons (40 mitochondria from each compartment). Both axonal and dendritic mitochondria in MICU1 OE showed faster depolarization than in control neurons. (f) Representative images showing cell viability before (pre-NMDA) and after NMDA stimulation. White arrows indicate cell bodies before NMDA treatment; blue arrows indicate surviving cells after stimulation; red arrows indicate cells that underwent cell death. Scale bar, 50 µm. (g) Quantification of cell death ratio under excitotoxic conditions (Control, 5 coverslips with 253 cells; MICU1 OE, 7 coverslips with 429 cells). Cell death percentage was significantly higher in MICU1 OE neurons. Data are presented as mean±SEM; bold lines indicate mean values, and shaded areas represent SEM. *$p < 0.05$, **$p < 0.01$, ***$p < 0.001$; n.s., not significant. Scale bar, 5 µm unless otherwise indicated.

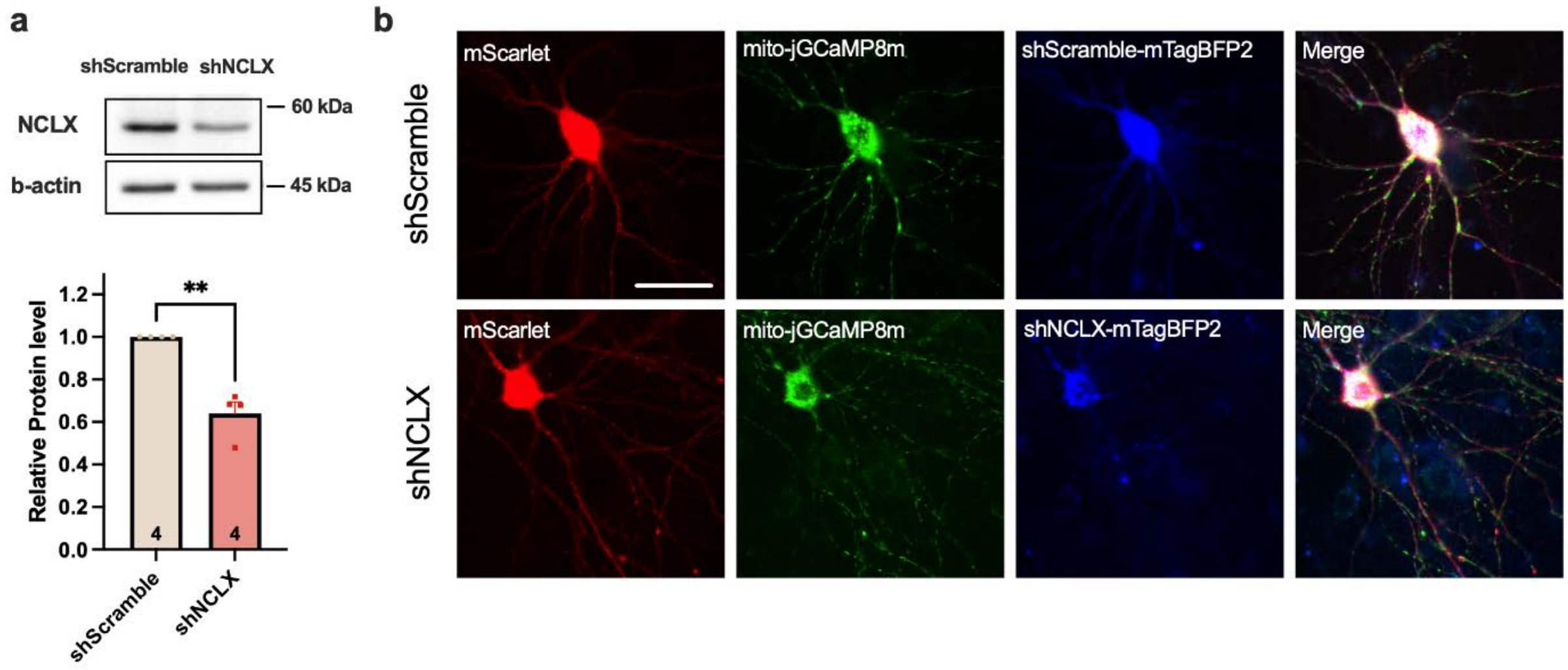

Supplementary Fig. 8. NCLX knockdown validation and representative images of recorded neurons. (a) Representative Western blot image from shScramble and shNCLX groups. Cortical neurons were infected by shScramble or shNCLX lentiviruses at 3-5 DIV and incubated for 10 days. Endogenous NCLX protein level is significantly reduced by NCLX knockdown. Relative protein level was normalized to values from the shScramble. (b) Representative images of recorded neurons. pCAG::mScarlet, pCAG::mito-jGCaMP8m, and pLKO-shScramble-mTagBFP2 or pLKO.1-shNCLX-mTagBFP2 were electroporated into the cortical pyramidal neuron. Paired t-test was used for bar graph in panel a. Sample numbers are annotated at the bottom of bar graph. Data are presented as mean±SEM. $**p < 0.01$. Scale bar, 50 μm

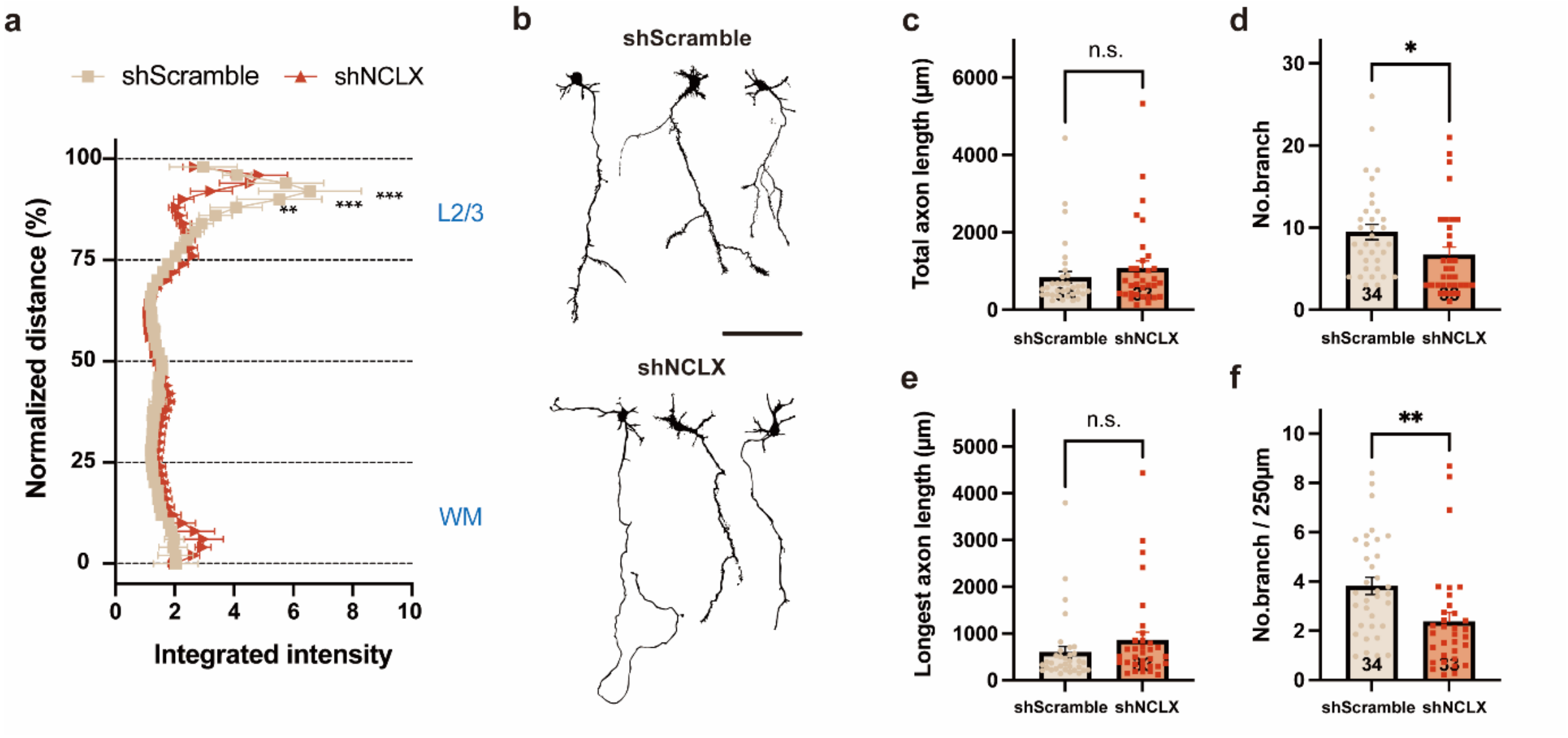

Supplementary Fig. 9. NCLX knockdown reduces axon branching in cultured cortical neurons.

(a) Quantification of integrated fluorescence intensity along the radial axis of the contralateral cortex reveals decreased contralateral callosal axon projection signal in layer 2/3 upon NCLX knockdown. Control n=4 mice, shNCLX n=5 mice. (b) Representative images of cultured cortical neurons expressing shScramble or shNCLX constructs at 5 DIV. Scale bar, 100 µm. (c–f) Quantifications of axonal morphology in shScramble and shNCLX neurons. (c) Total axon length. (d) Total branch number. (e) Longest axon length. (f) Number of branches per 250 µm. Data are presented as mean±SEM. *$p < 0.05$, **$p < 0.01$; n.s., not significant.

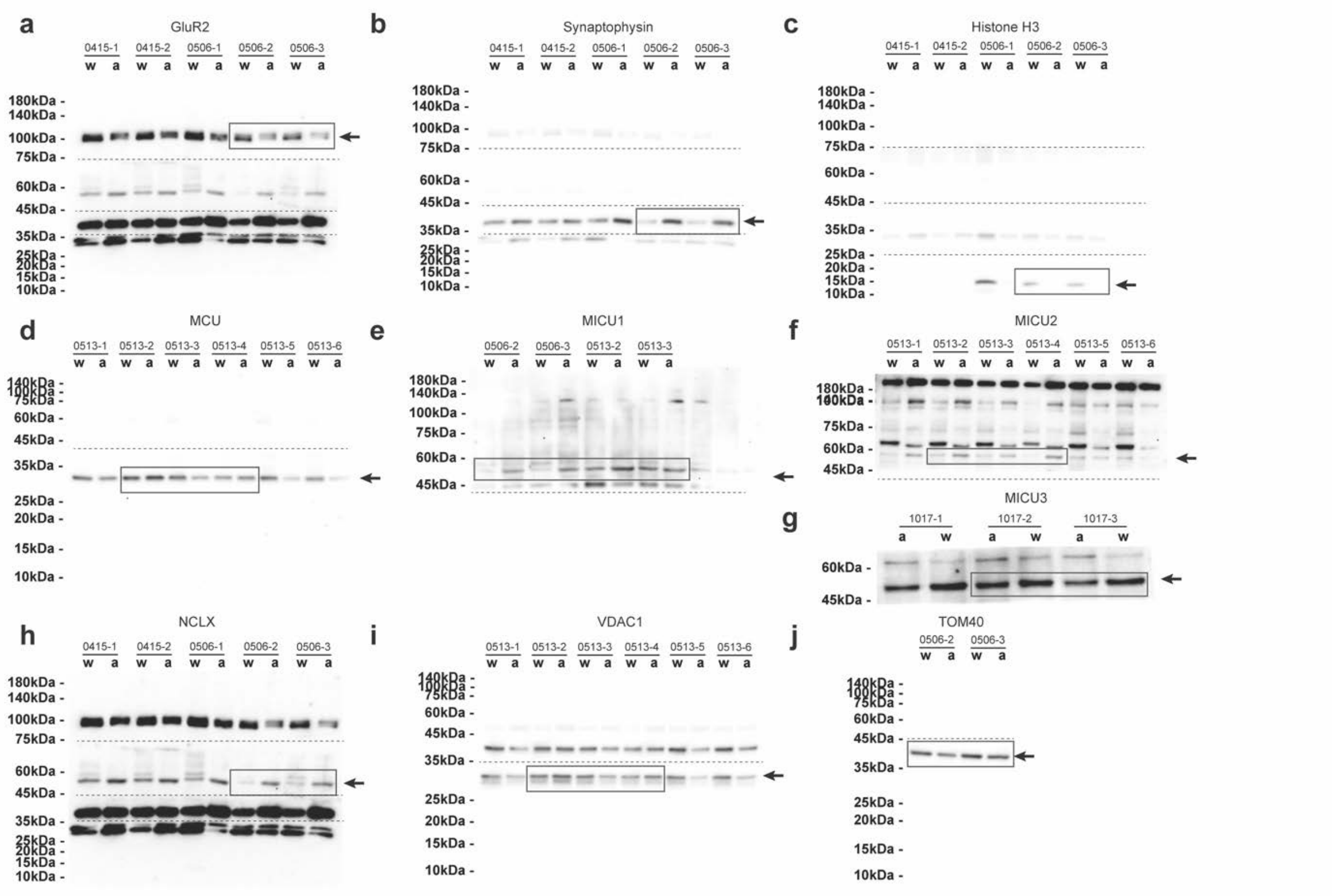

Supplementary Fig. 10. Full length Western blots of cortical neuron.

Raw blot images for Western blot experiments described in this study. Images show merged chemiluminescence and membrane signals. (a) GluR2, (b) Synaptophysin, (c) Histone H3, (d) MCU, (e) MICU1, (f) MICU2, (g) MICU3, (h) NCLX, and (i) VDAC1 corresponding to Fig. 2. (j) TOM40 corresponding to Supplementary Fig. 4. The lanes labeled with box indicate the samples used both in the main figure panels and for quantitative analyses. Unlabeled lanes correspond to samples not included in the final quantification because of incomplete fractionation. *w*, whole cell; *a*, axon.

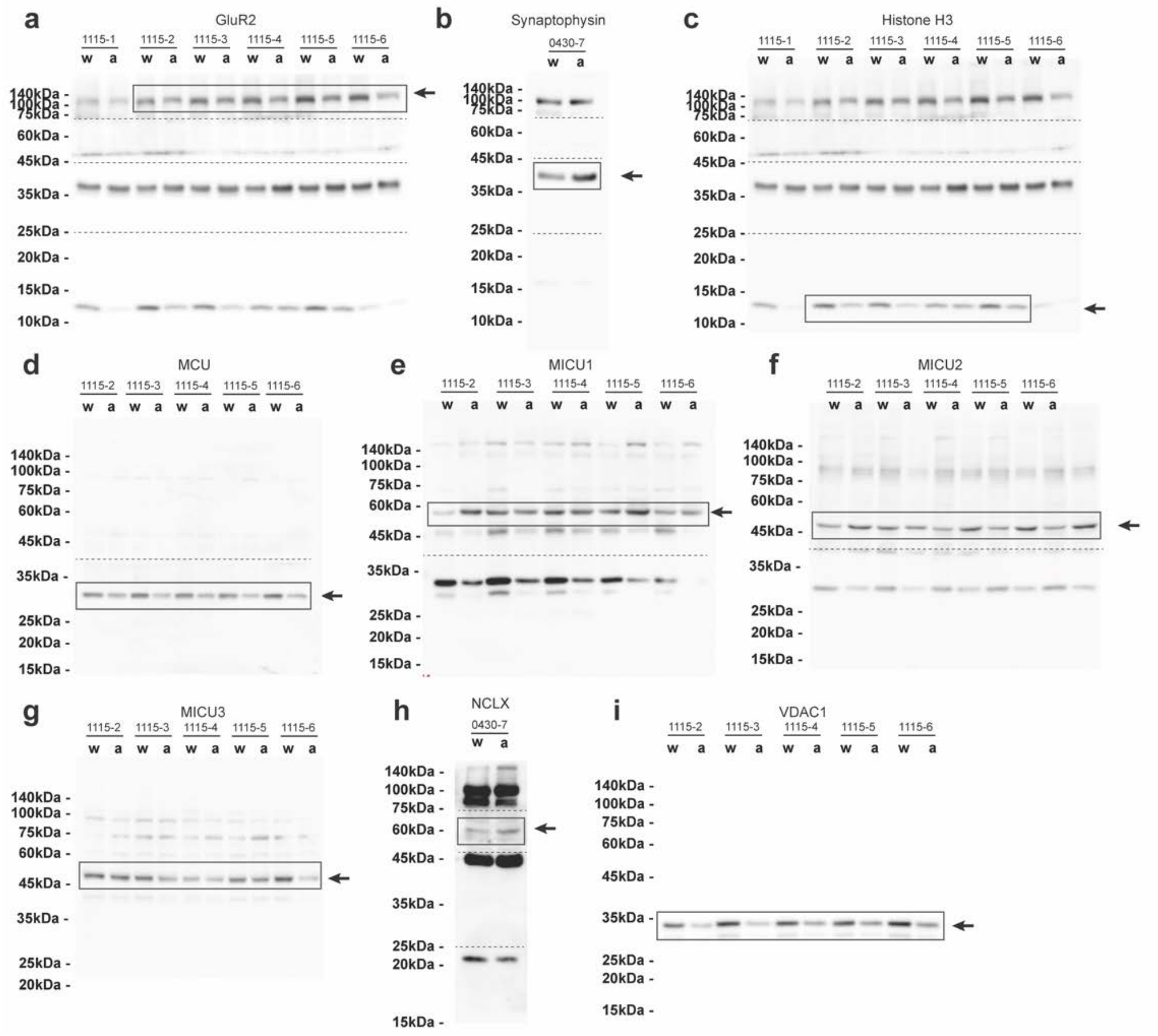

Supplementary Fig. 11. Full length Western blots of hippocampal neuron.

Raw blot images for Western blot experiments described in this study. Images show merged chemiluminescence and membrane signals. (a) GluR2, (b) Synaptophysin, and (c) Histone H3 corresponding to Supplementary Fig. 5. (d) MCU, (e) MICU1, (f) MICU2, (g) MICU3, (h) NCLX, (i) VDAC1 corresponding to Fig. 3. The lanes labeled with box indicate the samples used both in the main figure panels and for quantitative analyses. Unlabeled lanes correspond to samples not included in the final quantification because of incomplete fractionation. *w*, whole cell; *a*, axon.

Description of Additional Supplementary Files

Supplementary Video 1:
Ca^{2+} release in axonal and dendritic mitochondria

Supplementary Video 2:
Ca^{2+} uptake in axonal mitochondria.

Supplementary Video 3:
Ca^{2+} uptake in dendritic mitochondria.

Supplementary Video 4:
Ca^{2+} uptake after MICU1 or MICU2 overexpression in axonal mitochondria.

Supplementary Video 5:
Ca^{2+} uptake after MICU1 or MICU2 overexpression in dendritic mitochondria.

Supplementary Video 6:
NCLX knockdown delays Ca^{2+} release in axonal mitochondria

Supplementary Video 7:
NCLX knockdown did not affect Ca^{2+} release in dendritic mitochondria
